# Emotional impact of using different sensory modalities for autobiographical activation: a systematic review and meta-analysis

**DOI:** 10.3389/fpsyg.2025.1574855

**Published:** 2025-10-23

**Authors:** Dolores Fernández-Pérez, Beatriz Navarro-Bravo, Abel Toledano-González, Jorge Javier Ricarte, Jose Miguel Latorre-Postigo, Laura Ros

**Affiliations:** ^1^Research Institute in Neurological Disabilities, University of Castilla La Mancha, Albacete, Spain; ^2^Department of Psychology, Faculty of Medicine, University of Castilla La Mancha, Albacete, Castilla-La Mancha, Spain; ^3^Department of Psychology, Faculty of Health Sciences, University of Castilla La Mancha, Talavera de la Reina, Spain

**Keywords:** autobiographical memory, emotional induction, emotion regulation, emotion, mood

## Abstract

**Introduction:**

To the best of our knowledge, no previous work has synthesized the efficacy of MIPs based on the retrieval of AMs in relation to a wide range of both basic and complex emotions. This gap highlights the need to better understand how these interventions influence emotional states.

**Methodology:**

Accordingly, we conducted a systematic review of the literature and, when data were sufficient, a meta-analysis of pre-post changes in affective state. Our aim was to identify the most effective procedures for manipulating mood within empirical studies, and to develop a coherent framework to organize and interpret the efficacy of these types of MIPs, providing a foundation for future research.

**Results:**

The existing evidence is fragmented across heterogeneous protocols and lacks a unified quantitative synthesis. This fragmentation underscores the necessity of a systematic review and meta-analysis, which the present article undertakes to address.

**Discussion:**

Furthermore, we consider the growing interest in the field of “memory therapy”, given its clinical benefits in helping individuals access past events and allowing them to relive experiences and their associated emotions. This area holds promise for enhancing emotional regulation and therapeutic outcomes.

**Systematic review registration:**

https://www.crd.york.ac.uk/PROSPERO/view/CRD42021249072, identifier: CRD42021249072.

## 1 Introduction

Accessing Autobiographical Memory (AM) allows an individual to experience a subjective sensation of reliving a past event by means of a mental journey through time. The more vivid and richer the construction of the memory is, the greater will be this sensation (Suddendorf et al., 2009). It is worth noting that accessing AMs may involve the reincorporation of moods and mental states associated with the original event. These, however, need not necessarily be identical, just as the re-experiencing of the sensory, spatial, and perceptual experiences of the event need not be so, either (Wheeler et al., 1997; Conway, 2001). It has been suggested that emotion regulation is a distinctive function of AM (Harris et al., 2014), since autobiographical recall involves evoking memories from which to reactivate the emotions of the original emotional experience, accentuating their positive or negative details (Pascuzzi and Smorti, 2017). This is why access to AM has been used as an effective mood induction procedure.

In artificial laboratory situations, Mood Induction Procedures (MIPs) allow for the controlled and momentary study of emotions like those experienced in real situations. They permit the complexity of emotional processes to be analyzed, facilitating the study of the causal influence of emotions on different psychological and biological variables (Siedlecka and Denson, 2019). In recent years, several MIPs have been developed, with access to AM being one of the most widely used and effective (Siedlecka and Denson, 2019; Suardi et al., 2016), showing greater efficacy compared to other MIPs that do not use AM to generate emotions (e.g., simply listening to music or guided imagery) (Jallais and Gilet, 2010). This greater effectiveness appears to be because accessing AMs activates brain regions similar to those activated during the original emotional experience of the event remembered (Kober et al., 2008).

Access to AMs has been effectively employed in the induction of a wide range of emotions: anger (Siedlecka et al., 2015), disgust (Lane et al., 1997), surprise (Levenson et al., 1991), happiness and fear (Rainville et al., 2006), and sadness (Marci et al., 2007). Notably, it has been shown to be particularly effective in inducing positive mood states (Jallais and Gilet, 2010), even allowing for emotional recovery after the induction of negative affect (Öner and Gülgöz, 2018). Additionally, accessing AMs as an MIP is supported by a high level of ecological validity since the recall of past events is a frequent cause of emotional states in people's daily lives (Allen et al., 2014).

In MIPs based on access to AM, the participant is typically asked to freely recall, as vividly as possible, a past event with a certain emotional charge, such that they strive to re-experience the sensations, emotions, perceptions, and reactions of the original event (Westermann et al., 1996). To help individuals retrieve such events, different types of stimuli or cues can be used. The more information about the memory is present in the cue, the more immediate access to that memory will be (Uzer and Brown, 2017). Thus, the more accessible and meaningful the cues employed, the more effective the MIP will be.

Previous findings report that access is faster and more direct when the retrieval cue represents information that is relevant to a person's life, as well as when it contains much of the information about the event recalled (Willander et al., 2015). The most frequently used cues are verbal (e.g., keywords), followed by musical (excerpts from songs), olfactory (scents) and visual (e.g., images, film clips) (Fernández-Pérez et al., 2022). Furthermore, various studies have used unimodal cues (stimuli from a single sensory modality), while others have administered bimodal or multimodal cues (sensory stimuli from more than one modality).

To the best of our knowledge, no previous work has synthesized the efficacy of MIPs based on the retrieval of AMs and in relation to a wide range of both basic and complex emotions. Accordingly, we conducted a systematic review of the literature and, when data were sufficient, a meta-analysis of pre-post changes in affective state to quantify pooled effects. Our intention is to determine the most effective procedures when manipulating mood in the context of empirical studies and help create a coherent framework under which to organize the efficacy of these types of MIPs, and to provide the groundwork for future research. The existing evidence is fragmented across heterogeneous protocols and lacks a unified quantitative synthesis. Therefore, a systematic review and meta-analysis are needed; the present article undertakes that task. Furthermore, we consider the growing interest in the field of “memory therapy” (Dalgleish and Werner-Seidler, 2014), given its clinical benefits in helping individuals access past events and allowing them to relive experiences and their related emotions.

## 2 Methods

This study was conducted following the Cochrane Handbook for Systematic Reviews (https://handbook-5-1.cochrane.org/), and the PRISMA statement guidelines. The protocol used in this review was registered in the PROSPERO database (registration number CRD42021249072).

### 2.1 Search process

An exhaustive search was conducted in four of the most prominent databases related to our area of study: Scopus (Elsevier), PsycInfo (American Psychology Association), Web of Science and Pubmed (Medline). The following customized search string was used in full in the title, abstract and keyword fields: (“Autobiographical Memory”) AND (“Emotional Induction” OR “Emotion Regulation” OR “Emotion” OR “Mood”). The search results were imported into Covidence (https://www.covidence.org).

### 2.2 Eligibility criteria

This review included articles on access to AMs using different types of cues as the MIP. The following inclusion criteria were established:

- Empirical studies with adult participants (e.g., cross-sectional, quasi-experimental, and experimental studies) involving the use of autobiographical stimuli as MIP.
- Studies published before September 2025.
- Studies using samples of adults aged over 17 years. This age was established as most studies are conducted with university student populations.
- Studies in which the MIP included pre- and post-test measures to corroborate the effect of the induction procedure.
- Studies in which the MIP used standardized self-report instruments to measure the emotions to be elicited.

The exclusion criteria were as follows:

- Articles published in languages other than English or Spanish.
- Articles on systematic reviews and meta-analyses (although they were taken into account to avoid losing important references).
- Studies conducted with clinical population, and participants with depressive symptomatology or cognitive impairment.
- Studies using pharmacological treatments.

### 2.3 Selection of articles

**Figure 1:**
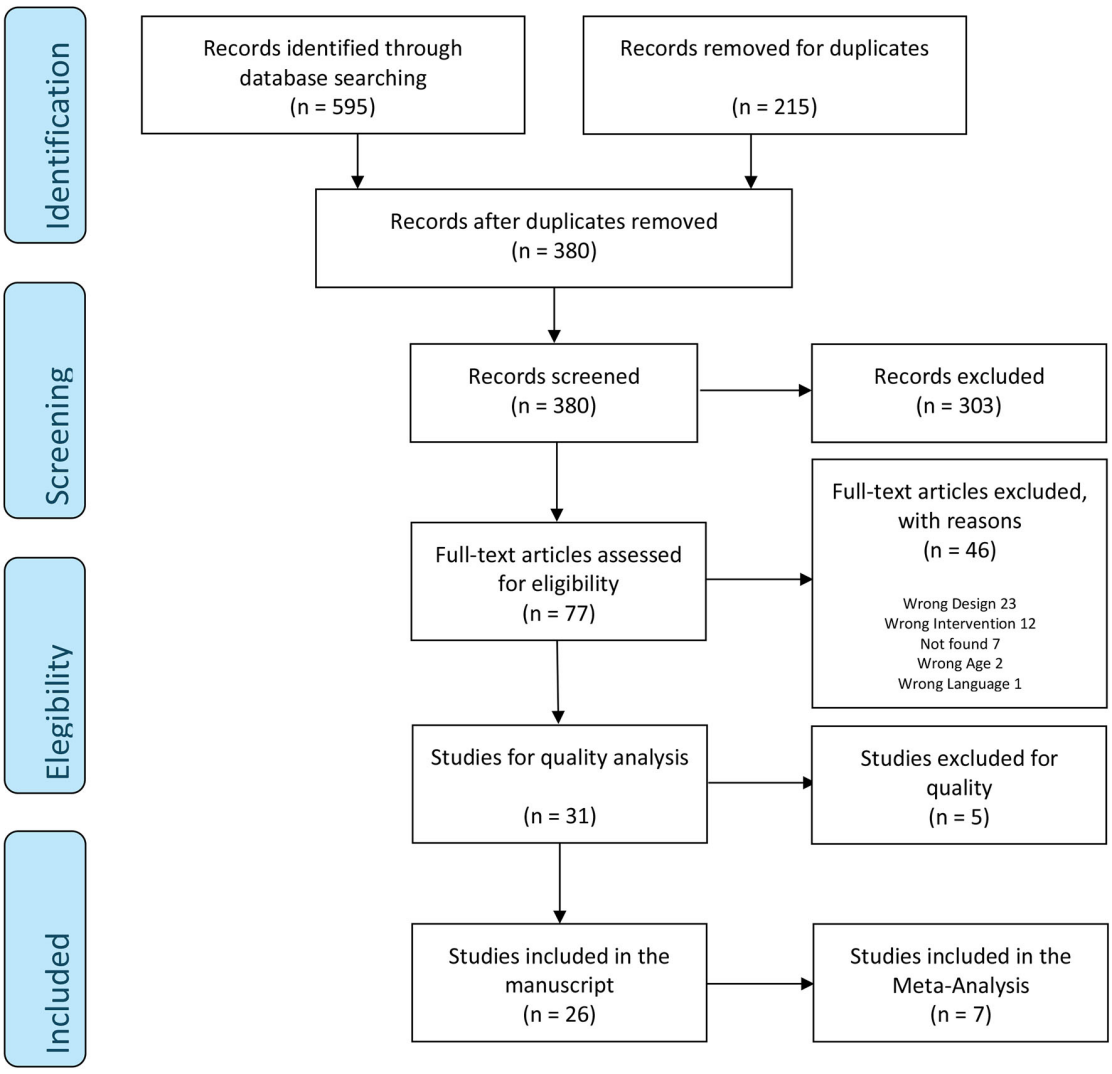
PRISMA 2009 flow diagram.

Figure 1 shows the flow chart summarizing the screening process. In the screening phase, 820 articles were obtained, of which Covidence eliminated 251 as duplicate papers, leaving 569 articles. These were evaluated through a two-step process: (1) screening of titles and abstracts and (2) full-text review. Two authors independently applied the established eligibility criteria to all the articles, screening titles and abstracts. All disagreements on inclusion/exclusion were judged by an independent third author.

Following this first selection phase, 475 articles were eliminated, leaving 94 works. In the next phase, two authors independently read the full text of these studies, with any discrepancies being resolved by the third author. This led to the elimination of 62 publications, leaving only 32 articles for the quality analysis, which, in turn, resulted in the discarding of 5 further articles. Consequently, 27 studies were selected for inclusion in the present systematic review. Given the variety and methodological differences in the studies, only 13 were considered for the meta-analysis. However, due to the lack of data, 4 of them had to be excluded, leaving a final total of 9 studies.

### 2.4 Quality analysis

The methodological rigor of the 32 studies was assessed using the Joanna Briggs Institute Critical Appraisal Checklist (JBC) (Jordan et al., 2019). We used the checklists corresponding to cross-sectional (8 items), quasi-experimental (9 items) and experimental (11 items) studies (see Table 1). The items contained in the different checklists can be rated as “Yes”, “No”, “Unclear” or “Not/Applicable”. Although this is a qualitative appraisal tool, for this review, and with the aim of determining as rigorously as possible the quality of the studies for inclusion/exclusion, a quantitative assessment was established whereby we considered high-quality studies to be those that were marked “yes” 10 or more times in the case of experimental studies, 5 or more times in the case of quasi-experimental studies (Bayes et al., 2019) and 6 or more times in the case of cross-sectional works (Burks et al., 2021). The quality analysis of each study was conducted independently by two authors and disagreements were resolved by discussion within the work team.

**Table 1:** Table summarizing the main characteristics of the studies included in the systematic review.

**Author (Year)**	**Sample size and characteristics**	**Aim**	**Instruments**	**Procedure**	**Outcome**
(Allen et al. 2014)	Experiment 1: *N =* 24 (8 men and 16 women) Age:19–36 Experiment 2: *N =* 24 (9 men and 15 women) Age: 20–35	To examine the impact of the recall of emotionally-valenced autobiographical memories on subsequent working memory performance.	Differential Emotions Scale (DES) (Izard et al., 1974; Schaefer et al., 2010)	Experiment 1: Participants were asked to retrieve an autobiographical memory of neutral or negative emotional valence as quickly as possible and in a vivid way. After this, they were asked to describe it orally for 1 min, emphasizing the details. Experiment 2: Participants followed the same guidelines as in experiment 1, including the autobiographical memory of positive, negative, and neutral valence.	Experiment 1: the multivariate effect of valence was significant *F*_(13, 312)_ = 20.44, *p <* 0.001, η^2^ = 96. Also, the univariate effect of valence was significant at *p <* 0.001, for joy, sadness, anger, guilt, and anxiety; at *p <* 0.010 for fearful and moved and of *p <* 0.050 for disgust, shame, and disdain. Negative recall led to the activation of negative emotions observing a change from *M* = 0.46(0.02) to *M* = 0.54(0.03). Experiment 2: the multivariate effect of valence was significant *F*_(26, 312)_ = 7.94, *p <* 0.001, η^2^ = 0.75. Additionally, the univariate effect of valence was significant at *p <* 0.001, for joy, sadness, anger, fearful, loving and moved; at *p <* 0.010 for anxiety, disdain, surprise, shame and guilt and of *p <* 0.050 for disgust. Positive recall led to activation of positive emotions, observing a change from *M* = 0.47(0.02) to *M* = 0.72(0.02), just as negative recall led to activation of negative emotions with a change from *M* = 0.44(0.03) to *M* = 0.71(0.02).
(Barrett and Janata 2016)	*N =* 12 (4 men and 8 women) Age: 19–33	To identify brain regions involved in affective and mnemonic processes using a music-evoked AM task.	Southampton Nostalgia Scale (SNS) (Sedikides et al., 2008) Affective Neuroscience Personality Scale (ANPS) (Davis et al., 2003; Davis and Panksepp, 2011)	Thirty musical excerpts (20 s.) of songs from when the participants were aged between 7 and 19 years old were presented and after each excerpt the participant was asked to rate the degree to which the stimulus led them to experience nostalgia, happiness, sadness, as well as autobiographical salience, arousal, and familiarity with the stimulus. The time set between excerpts was 5 seconds. During the entire course of the experiment, music listening, resting state, and emotion tasks were completed while the blood oxygenation level-dependent signal was recorded on an MRI scanner.	We found that scores on brain activity during music-evoked nostalgia were highly correlated with ratings of familiarity with the stimulus (r = 0.787, *p <* 0.001) and autobiographical salience (r = 0.782, *p <* 0.001).
(Benau and Atchley 2020)	*N =* 31 (18 men and 13 women) Age: 18–31	To study how the transient changes in mood (sadness) influence the perception of time in neutral stimuli.	Visual Analog Scale (VAS) (Hayes and Paterson, 1921; Rossi and Pourtois, 2012)	Participants listened to Sergei Prokofiev's “Russia under the Mongol Yoke” at half speed (6 min) twice. Meanwhile, they were asked to think of a sad autobiographical memory in as much detail as possible and then write it down on paper. Each listening was separated by 1 second.	There was a main effect of Time, *F*_(2, 60)_ = 7.23, *p* = 0.001, η^2^ = 0.194, and Emotion *F*_(3, 90)_ = 52.94, *p <* 0.001, η^2^ = 0.638, as well as a significant interaction Emotion × Time, *F*_(6180)_ = 17.93, *p <* 0.001, η^2^ = 0.374. Happiness was significantly lower after induction (*p <* 0.001, *d* = 1.24) or at the end of the experiment (*p* = 0.032, *d* = 0.63), and lower at the end of the experiment than at the beginning (*p* = 0.046, *d* = 0.61). Sadness was higher after induction than at the beginning (*p <* 0.001, *d* = 1.25) or at the end of the study (*p <* 0.001, *d* = 1.32), and the level of sadness at the end and at the beginning did not differ significantly (*p* = 1.0, *d* = 0.07). Participants reported greater fear after induction than at the end of the experiment (*p* = 0.008, *d* = 0.14). Fear levels at the beginning of the experiment did not differ significantly after induction or at the end of the study (*p* > 0.154). Anger levels were similar between study stages (*p*>0.094). As a result of induction, an increase in negative emotions and a decrease in positive emotions were observed, with the greatest change observed in the emotion of happiness [*M* = 4.43(3.4)], followed by sadness [*M* = 4.25(3.2)], anger [*M* = 1.91(2.3)] and fear [*M* = 1.34(1.9)]. The difference between the scores before and after induction for sadness correlated negatively with those for happiness (r = −0.596, *p <* 0.001) and positively with fear (r = 0.434, *p* = 0.015) and anger at the trend level (r = 0.316, *p* = 0.083).
(Burns et al. 2003)	*N =* 64 (32 women and 32 men) Age M = 25.1(3.4)	To examine the relationship between anger management style and subsequent pain sensitivity.	McGill Pain Questionnaire-Short Form (MPQ) (Melzack, 1987) Positive and Negative Affect Scale (PANAS) (Watson et al., 1988) Anger Expression Inventory (AEI) (Spielberger et al., 1985)	Participants were randomly assigned to each of three conditions (anger, sadness, and joy). They then took part in semi-structured interviews in which they were asked to recall and describe a past (albeit recent) event that elicited the assigned emotion.	For PANAS PA scores there was a significant Time x Condition interaction *F*_(2, 61)_ = 11.40, *p <* 0.001. Results showed that AP significantly increased from baseline for the joy condition *F*_(1, 20)_ = 12.04, *p <* 0.002, while they decreased for the sadness condition *F*_(1, 20)_ = 7.48, *p <* 0.001. No changes were perceived for the anger condition *F*_(1, 20)_ = 3.18, *p <* 0.09. For PANAS NA scores the Time x Condition interaction was also significant *F*_(2, 61)_ = 19.03, *p <* 0.001. The results showed that NA significantly increased from baseline for the anger condition *F*_(1, 20)_ = 26.38, *p <* 0.001, and sadness *F*_(1, 21)_ = 22.92, *p <* 0.001. In joy, however, it significantly decreased *F*_(1, 20)_ = 11.55, *p <* 0.005.
(Cady et al. 2008)	*N =* 124 (61 women and 63 men) Age: 18–22	To examine the relationship between popular music and autobiographical memory.	Positive and Negative Affect Scale (PANAS) (Watson et al., 1988) 5-point Likert-type scale for vividness, specificity, reliving, degree of pleasantness of the memory and intensity of the predominant emotion.	Based on a pilot study, songs were chosen from five different eras: early childhood, grade school, middle school, high school, and college (5/10 songs per era). The participant was shown the list of songs for each era and asked to choose the song with the strongest associated positive autobiographical memory. Participants were then randomly assigned to one of 4 conditions: one control condition and 3 experimental conditions. In the control condition, they were shown only the title of the song; in the audio condition, they listened to the chosen song for 1 min; in the visual condition, they were shown the lyrics written on a page; and in the picture condition; they were shown the album cover. When the stimulus was presented, they were asked to recall and write about the associated memory in as much detail as possible (rating vividness and specificity and predominant emotion). All participants performed the task 5 times (once for each era) but within the same sensory condition.	Overall, participants' PA did not change, with this pattern being maintained for each of the conditions. The mean PA score for all participants was 2.74 out of a possible 5.00 (0.73) before the experiment and 2.78 (0.74) after the experiment. The mean NA for all participants were 1.34 (0.38) before the experiment and 1.25 (0.35) after the experiment. Participants' NA decreased and *t*-test results were significant, *t*_(123)_ = 2.78, *p <* 0.050, although this difference occurred in only two of the four groups and was small in magnitude. Those in the auditory condition showed a significant decrease in NA [*t*_(30)_ = 3.17, *p <* 0.050] as did those in the imagery condition [*t*(30) = 2.25, *p <* 0.050], while the print group and control showed no change.
(Carretero et al. 2020)	*N =* 40 Age M = 77.21(7.79)	To analyze the effects of autobiographical stimuli on emotion regulation in older adults.	Self-Assessment Manikin (SAM) International Affective Picture System (IAPS) (Lang et al., 2008)	Participants were assigned to two conditions (IAPS and personal photos) Initially, participants undertook a negative mood induction by viewing a 7-min movie scene (“Dead Man Walking”). This was followed by the mood recovery task by means of a positive emotional induction. For this purpose, participants in both conditions (IAPS and personal photos) were asked to generate a specific positive autobiographical memory from the visualization of 5 images (personal or impersonal depending on the group).	A significant main effect of Time *F*_(2, 36)_ = 261.29, *p <* 0.001, η^2^ = 0.94 was observed. Participants felt significantly more pleasant after the mood recovery task compared to before the induction in both conditions *F*_(1, 37)_ = 24.65, *p <* 0.001, η^2^ = 0.40 as well as after negative emotional induction *F*_(1, 38)_ = 484.26, *p <* 0.001, η^2^ = 0.93. For emotional arousal there was a significant main effect of Time *F*_(1, 37)_ = 11.76, *p* ≤ 0.001, η^2^ = 0.24. In both conditions participants rated themselves as less aroused after the mood recovery procedure than before it, *F*_(1, 38)_ = 59.19, *p* ≤ 0.001, η^2^ = 0.61, and showed greater dominance after the mood retrieval procedure, *F*_(1, 38)_ = 73.30, *p <* 0.001, η^2^ = 0.66. Also, no differences were observed between groups with respect to nostalgia scores and sense of reliving the memory. A positive correlation was found between sense of reliving and pleasant feeling in the IAPS group (r = 0.49, *p* = 0.023).
(Dehghani et al. 2022)	*N =* 35 (100% male) Control Group: *N =* 15 Age M = 26.7(3.6) Experimental Group: *N =* 20 Age M = 27(3.8)	To investigate how EEG Neurofeedback changes EEG and fMRI BOLD signals, psychometric assessments, and connectivity in brain regions during an emotion regulation process.	Positive and Negative Affect Scale (PANAS) (Watson et al., 1988) Persian Version of Beck Anxiety Inventory (BAI) (Beck et al., 1988a,b) The State Trait Anxiety Inventory (STAI) (Azizi et al., 2013) Short Persian Version of the Profile of Mood States (POMS) (Spielberger, 1972)	The study had two experimental groups that differed in the type of neurofeedback. All participants were asked to describe several happy autobiographical memories before the start of the experiment. After this, the participants had to generate these memories through interviews with the help of images whose subject matter was in accordance with the content of the memories. The experiment involved the following sequence: 1) rest, 2) task of visualization of two images of the 1940s but without generation of autobiographical memories or thoughts about them, 3) task of emotion regulation through autobiographical memories. In the experimental group, the recall was accompanied by neurofeedback based on increasing or maintaining the height of a bar based on information from the brain activity itself. In the control group the neurofeedback was a randomly generated signal. Participants' mood was measured before and after the experimental task.	The results confirm the efficacy of neurofeedback in the experimental group. The mean scores of PANAS did not change significantly due to neurofeedback, but the changes in PANAS positive and negative mood: [*t*_(17)_ = 4.01, *p <* 0.001] and [*t*_(17)_ = 3.32, *p <* 0.001] respectively are significant. Also, in the POMS and Total Mood Distribution (TMD), the changes are significant [*t*_(17)_ = 3.61, *p <* 0.001] and [*t*_(17)_ = 3.31, *p <* 0.001]. This demonstrates that neurofeedback is effective in increasing positive mood state and decreasing negative mood state by recalling positive autobiographical memories. For the control group, negative mood PANAS scores are significant (paired t; gl = 13; *p* = 0.064) but for positive mood states, they do not change significantly. This demonstrates that positive autobiographical recollection, even with simulated neurofeedback, is effective in decreasing the negative mood of the control group. POMS and TMD scores did not change significantly. Anxiety also decreased overall in all participants with scores on the BAI from *M* = 8(4.4) to *M* = 6.6(4.7) and on STAI from *M* = 27(2.5) to *M* = 26.3(2.8).
(Fabiansson et al. 2012)	*N =* 21 (11 women and 10 men) Age M = 21	To analyze the brain regions activated during reappraisal, analytical rumination and anger rumination involved in cognitive control and reward.	Mood Adjective CheckList (MACL) (Nowlis, 1965)	Participants were asked to recall a past event (within the last 12 months) associated with the emotion of anger and to rate how they felt at the time of the original event and at the time of recall by completing the MACL and rating it in terms of vividness, intensity and distress. In a subsequent session, participants were induced in the emotion of anger using the memory they generated in the previous session. After this, they were exposed to three contrasting ER conditions (reappraisal, analytic rumination, and anger rumination) each of which instructed the participant how to think about the anger-inducing memory. Upon completion of the three conditions, participants again completed the MACL.	A significant interaction was identified between ER strategy during each of the 3 conditions *F*_(4, 80)_ = 40.07, *p <* 0.001, *d* = 2.78; reporting greater reappraisal when they were instructed to engage in analytical rumination *t*_(20)_ = 5.09, *p <* 0.001, *d* = 1.14 or anger *t*_(20)_ = 8.09, *p <* 0.001, *d* = 1.77; on the other hand they engaged in more analytical rumination during the analytical condition than during the reappraisal *t*_(20)_ = 4.28, *p <* 0.001, *d* = 0.93 or anger condition *t*_(20)_ = 6.25, *p <* 0.001, *d* = 1.38; during the anger condition, more anger rumination was conducted than during the reassessment *t*_(20)_ = 8.70, *p <* 0.001, *d* = 1.90 or the analytic condition *t*_(20)_ = 7.93, *p <* 0.001, *d* = 1.78.
(Fernández-Pérez et al. 2023)	*N =* 263 Youth: *N =* 139 (67.6% female) Age M = 19.57(1.86) Older: *N =* 124 (68.5% female) Age M = 72.26(5.59)	To analyze the efficacy of different types of imagery in eliciting specific positive autobiographical memories with the aim of achieving mood enhancement after a negative emotional induction.	Positive and Negative Affect Scale (PANAS) (Watson et al., 1988) International Affective Picture System (IAPS) (Lang et al., 2008)	The study had three experimental conditions according to image type (IAPS, place and personal). Young and older participants were randomly assigned to each condition. Prior to the experimental task, participants were asked to select a total of 6 images (according to condition) associated with specific positive autobiographical memories. The experimental phase began with a negative mood induction by viewing a 7-min movie clip. This was followed by a positive mood induction in which participants were asked to observe each of the previously selected images associated with specific positive autobiographical memories. Participants were asked to concentrate on the memory and try to relive the emotions associated with it. Before and after both positive and negative mood induction, participants were asked to complete the PANAS.	The results showed an increase in positive affect [*F*_(1, 254)_ = 17.83, *p <* 0.001, η^2^ = 0.07] of 30.5%, as well as a decrease in negative affect [*F*_(1, 254)_ = 31.00, *p <* 0.001, η^2^ = 0.11] of 46.9%. Regarding the decrease in negative affect, the efficacy of personal photographs [*F*_(1, 254)_ = 4.10, *p <* 0.050, η^2^ = 0.03] stood out with a 50% change, followed by those of locations (46.7%) and IAPS (43.9%). Also in the decrease of negative affect, differences were found between age groups [*F*_(2, 254)_ = 4.77, *p <* 0.050, η^2^ = 0.04], such that in the older ones IAPS images worked better with a decrease of 45.9% and in the younger ones personal photographs with a decrease of 53.84%.
(Gendolla et al. 2001)	*N =* 42 (29 women and 13 men) Age M = 23	To demonstrate that relatively long-lasting mood states do not involve effort-related autonomic adjustments but may affect effort mobilization when experienced in the context of demands whose outcome is controllable.	UWIST Mood Adjective Checklist (Matthews et al., 1990)	Participants were instructed on the task, which would last for 10 min. Participants in the negative mood music condition group listened to one piece of sad music. Participants in the positive mood music condition listened to four pieces of happy and easy-listening music. In the autobiographical recall conditions, participants, according to condition, had to retrieve and describe a positive or negative life event (poignant and important life events) by recalling and describing it in a vivid and emotional way. The memories were stored in an envelope.	Mood states were successfully manipulated *F*_(1, 38)_ = 4.59, *p <* 0.04, both positive (*M* = 0.26, *SE* = 0.13) and negative (*M* = −0.36, *SE* = 0.26) mood.
(Gendolla et al. 2005)	*N =* 46 (24 women and 22 men) Age M = 23	To study whether self-focused attention promotes the experience of symptoms when people are in a certain positive or negative mood state.	UWIST Mood Adjective Checklist (Matthews et al., 1990)	Participants were first asked to assess their mood using the UWIST. After this, participants were instructed to recall and describe a positive or negative life event in a vivid and emotional way. While recalling, participants were given either a self-focus manipulation in which they had to remain in front of a mirror or a non-self-focus manipulation in which participants remained seated in front of a wall. At the end, participants were asked to complete the UWIST again.	The results showed successful manipulation of successful mood (*F*_(1, 42)_ = 8.88, *p <* 0.005) in both positive mood (*M* = 1.30, *SD* = 3.20) and negative mood (*M* = 3.00, *SD* = 6.25). There were no significant differences according to the self-focused and non-self-focused conditions.
(Gotz et al. 2022)	*N =* 17 (6 women and 11 men) Age: 21–57 yrs. Age M = 28.47(8.58)	To investigate how the methods of emotion induction through autobiographical recall used in the real-world work in Virtual Reality.	Self-Assessment Manikin (SAM) (Bradley and Lang, 1994)	Four methods of autobiographical recall through VR were developed from existing techniques: 1) speaking aloud to a virtual agent; 2) speaking to an avatar as a proxy for the researcher (i.e., the participant believes that avatar and researcher are connected); 3) thinking silently about past experience; and 4) writing/drawing using a VR pen. Each participant was required to complete four autobiographical recall tasks each using a different VR method. Participants were asked to recall four events from their life one that evoked happiness, one that evoked sadness, one that evoked anger, and one that evoked fear, in as much detail as possible. To measure the efficacy of the emotion induction, participants completed the SAM before and after each experimental task.	There were no statistically significant differences between the different methods on the valence (*p* = 0.91), arousal (*p* = 0.60) and dominance (*p* = 0.71) dimensions. There were no significant differences between any of the SAM dimensions on any of the emotions (*p* > 0.05). All methods were effective in inducing the target emotion.
(Jallais and Gilet 2010)	*N =* 160 (145 women and 15 men) Age M = 19.4 (3.47)	To analyze the effectiveness of two mood induction procedures (MIPs) to induce 4 specific mood states that vary according to valence and arousal level.	Brief Mood Introspection Scale (BMIS) (Mayer and Gaschke, 1988; Corson and Verrier, 2007) Adaptation of the Affect Grid (Russell et al., 1989)	Participants were randomly assigned to 8 possible conditions, with different induction targets (happy, serene, angry, and sad) and according to 2 types of procedure (autobiographical recall and music+guided imagery). In the autobiographical memory condition, participants were asked to recall and write down an event in their lives in which they had felt happiness, sadness, serenity or anger (depending on the condition) with as much vivid detail as possible. They were given a maximum of 10 min to do so. In the combined procedure condition, participants listened to 4-min of musical pieces (inducing an emotion according to the condition). After this, the experimenter performed a guided visualization task by describing everyday situations related to the target emotion	According to the group, scores significantly increased compared to baseline mood in the autobiographical recall groups. In the happiness group happiness increased *F*_(1, 71)_ = 7.27, M = 0.48, *p <* 0.001, η^2^ = 0.09 and anger decreased *F*_(1, 71)_ = 5.82, *M* = 0.45, *p <* 0.050, η^2^ = 0.08; in the serenity group, scores remained stable; in the sadness group, sadness increased *F*_(1, 71)_ = 27. 54, *M* = 0.58, *p <* 0.001, η^2^ = 0.28; and in the anger group, sadness and anger increased *F*_(1, 71)_ = 4.27, *M* = 0.58, *p <* 0.050, η^2^ = 0.06, with a decrease in happiness *F*_(1, 71)_ = 26.34, *M* = 0.48, *p <* 0.001, η^2^ = 0.27 and serenity *F*_(1, 71)_ = 21.04, *M* = 0.73, *p <* 0.001, η^2^ = 0.23. Arousal in the happiness and anger groups increased following the induction *F*_(1, 71)_ = 7.54, *M* = 1.06, *p <* 0.001, η^2^ = 0.09 and *F*_(1, 71)_ = 7.15, *M* = 1.06, *p <* 0.001, η^2^ = 0.09, respectively.
					In the combined procedure groups, the happiness group increased its serenity score *F*_(1, 81)_ = 5.75, *M* = 0.45, *p <* 0.050, η^2^ = 0.07; in the serenity group, there was an increase in serenity *F*_(1, 81)_ = 6. 62, *M* = 0.46, *p <* 0.050, η^2^ = 0.08; in the sadness group sadness increased *F*_(1, 81)_ = 8.99, *M* = 0.38, *p <* 0.010, η^2^ = 0.10; in the anger group anger increased *F*_(1, 81)_ = 30.47, *M* = 0.013, *p <* 0.001, η^2^ = 0.27. Arousal in the sadness and serenity groups decreased following the induction *F*_(1, 81)_ = 10.34, *M* = 1.63, *p <* 0.001, η^2^ = 0.11 and *F*_(1, 81)_ = 12.87, *M* = 1.63, *p <* 0.001, η^2^ = 0.14, respectively.
(Laco et al. 2021)	*N =* 106 (18 women and 88 men) Age:19–36	To examine the effect of mood state on attention.	PANAS Short-Form (I-PANAS-SF) (Karim et al., 2011; Watson et al., 1988).	The study had three conditions. In the happy autobiographical memory condition, participants were asked to recall a very happy event from their lives, imagining it as vividly and specifically as possible. Questions were used to guide the retrieval. In the neutral autobiographical memory condition, participants were asked to remember the route they had taken to go to the place the experiment was conducted at, including as many details as possible. In the happy music condition, the participants listened to Vivaldi's “The Four Seasons”.	Following the mood induction, the participants in the happy autobiographical memory group, were found to be significantly happier than those in the neutral condition group (*U* = 610, *p <* 0.050) and those in the music condition group (*U* = 250, *p <* 0.010). The music condition group was more enthusiastic than the neutral group (*U* = 312, *p* = 0.018) and the autobiographical memory group (*U* = 170, *p <* 0.001).
(Lievaart et al. 2017)	*N =* 72 (54 women and 18 men) Age: 18–23	To broaden the knowledge on the impact of angry rumination on self-control.	Visual Analog Scale (VAS) (Hayes and Paterson, 1921; Rossi and Pourtois, 2012) Positive and Negative Affect Scale (PANAS) (Watson et al., 1988)	The procedure began with a 5-min session to describe 3 negative events and indicate the degree of anger (moment of the event and degree of resolution). One of the events with the greatest induction was selected to be worked on in a 5-min semi-structured interview. In this interview, an attempt was made to relive the memory. After this, participants were assigned to one of two experimental conditions: angry rumination or distraction. In the angry rumination condition, they were asked to think about the anger-inducing memory and focus on the emotional aspects, while those in the distraction condition were asked to think about what the campus looked like (buildings, routes, things to do...).	Main effect of Time, *F*_(1, 70)_ = 133.48, *p <* 0.001, η^2^ = 0.66, showing that participants were angrier after anger induction (*M* = 35.81, *DT* = 21.12) than at baseline (*M* = 7.81, *DT* = 10.21)
(López-Cano et al. 2020) Study 1	*N =* 30 (66% women) Age: 35–80 Middle-aged adult group: *N =* 15 Age: 35–59 Older adult group: *N =* 15 Age: 63–80	To assess the capacity for mood-induction and autobiographical memory retrieval of songs from “different cultures” and from two different life periods in middle-aged and older adults.	Positive and Negative Affect Scale (PANAS) (Watson et al., 1988) Self-Assessment Manikin (SAM) (Bradley and Lang, 1994)	The stimulus used was a collection of songs from the pop charts (that had reached #1), one from each year between 1952 and 2017. A total of 130 thirty-second excerpts were used. Each participant listened to 10 song excerpts corresponding to the first 5 years of their Reminiscence Bump and 10 from the five years immediately following that period. The approximate standard Reminiscence Bump period was age 15–30 years.	There was a significant increase in PANAS PA in both age groups: middle-aged *t*_(14)_ = −3.20, *p* = 0.006, *d* = −0.28; older *t*_(14)_ = −5.29, *p <* 0.001, *d* = −0.69, with a larger effect in the older adult group. In PANAS NA, a significant decrease was observed in both groups with a larger effect size in older adults *t*_(14)_ = 4.60, *p <* 0.001, *d* = 1.29, compared to middle-aged *t*_(14)_ = 3.13, *p* = 0.007, *d* = 0.97. The retrieval of autobiographical memories generated a significant increase in subsequent mood R2 = 0.83 (*F* = 66.19, *p <* 0.001). In older adults, PA showed a significant increase, *t*_(14)_ = −5.29, *p <* 0.001, *d* = −0.69; while NA showed a significant decrease, t_(14)_ = 4.60, *p <* 0.001, *d* = 1.29). Impact of memory on mood: R2 = 0.83 (*F* = 66.19, *p <* 0.001) with *B* = 1.01 for the pre-test PA value (ß = 0.89, *t* = 11.24, *p <* 0.001, 95% CI 0.82–1.19) and with *B* = 0.03 for total number of memories (ß = 0.16, *t* = 2.08, *p* = 0.04, 95% CI 0.82–1.19). There was a significant increase in PA with a medium-low effect size, *t*_(34)_ = −2.11, *p* = 0.042, *d* = −0.30 and a significant decrease in NA with a large effect size, *t*_(34)_ = 4.72, *p <* 0.001, *d* = 0.80.
(López-Cano et al. 2020) Study 2	*N =* 35 (62.9% women) Age: 61–73	To assess the capacity for mood induction and autobiographical memory retrieval of songs from “different cultures” and from two different life periods in middle-aged and older adults.	Positive and Negative Affect Scale (PANAS) (Watson et al., 1988) Self-Assessment Manikin (SAM) (Bradley and Lang, 1994)	The stimuli used were songs taken from the pop charts from the years 1959 to 1991. 64 thirty-second excerpts (50% native popular music and 50% international popular music). Participants were exposed to 20 randomly selected pop songs and, after each excerpt, were asked questions associated with the variables under study (emotions, song familiarity and autobiographical recall). In the autobiographical recall, participants were asked to provide a detailed description of the autobiographical event (date and place where it occurred), being given a maximum of 5 min for each memory associated with each musical excerpt.	A significant increase in PANAS PA was observed with a medium-low effect size, *t*_(34)_ = −2.11, *p* = 0.042, *d* = −0.30 and a significant decrease in PANAS NA with a large effect size, *t*_(34)_ = 4.72, *p <* 0.001, *d* = 0.80.
(MacKinnon et al. 2013)	*N =* 30 (21 women and 9 men) Age: 23–35	To analyze heartbeat evoked potentials to examine cardiac regulation of afferents during emotion induction and resonant breathing.	Positive and Negative Affect Scale (PANAS) (Watson et al., 1988)	All participants took part in the two induction conditions: happiness and sadness. The induction was implemented by asking participants to evoke two emotionally significant autobiographical events that had produced sadness and happiness and to write them down, following the autobiographical script technique. Subsequently, the experimenter helped the participants mentally visualize the previously described autobiographical events. This lasted 5 min, during which the participants remained in silence with their eyes closed.	The PANAS scores were consistent with the data collected in a neutral condition from a non-clinical sample. The mean score for the PANAS PA *M* = 28.75 (*SD* = 4.8), with the norm being *M* = 30.62 (*SD* = 7.9), and *M* = 15.00 (*SD* = 4.4) for the PANAS NA, with the norm being *M* = 14.00 (*SD* = 5.9).
(Monno et al. 2024) Study 2	*N =* 141 (66 women and 75 men) Age M: 21.9 (1.53)	To analyze the effectiveness of implementing an online induction method for young adults	Visual Analog Scale (VAS) (Hayes and Paterson, 1921; Rossi and Pourtois, 2012) Positive and Negative Affect Scale (PANAS) (Watson et al., 1988) Positive and Negative Affect Scale (PANAS-X) (Kawahito et al., 2011)	All participants took part in both the happiness and sadness induction. Data was collected at four points during the experiment: at the beginning, after the mood induction, after the task, and after the washback session. Participants had to listen to each of the audio clips, after which sentences were displayed on the screen for the assigned condition and they had to associate them with a personal autobiographical memory. Each sentence was displayed for 12 s, and there were a total of 60 sentences.	For positive PANAS PA affect, a significant effect was found between time F_(2, 258)_ = 28.40, *p <* 0.001, η^2^ = 0.180 and the interaction between mood induction and time F_(8, 258)_ = 3.65, *p <* 0.001, η^2^ = 0.102 For negative affect PANAS NA, a significant interaction was found between mood and time F_(1.90, 245.38)_ = 4.33, *p <* 0.001, η^2^ = 0.118. Following the interaction, the PANAS PA score was significant F_(1.59, 38.14)_ = 8.34, *p <* 0.002, η^2^ = 0.258 For the PANAS-X scores, significant scores were shown in the effect of time F_(1.92, 247.39)_ = 25.30, *p <* 0.001, η^2^ = 0.164
(Nourkova and Gofman 2022)	*N =* 99 (68 women and 31 men) Age M = 20.31(2.06)	To analyze the influence of deliberately retrieved negative autobiographical memories in young adults.	Positive and Negative Affect Scale (PANAS) (Watson et al., 1988) Implicit Positive and Negative Affect Test (IPANAT) (Quirin et al., 2009) Zimbardo Time Perspective Index (ZPTI) (Zimbardo and Boyd, 1999)	The participants attended a total of 4 sessions beginning with the measurement of emotional state with a duration of 20 min. Once divided into 2 working groups, they started the memory task assigned according to group and, at the end, they completed the questionnaire again. Eight non-recurrent lists of stimuli were used where they were confronted with new sets of nonsense words. The questionnaire was administered via Google.	Significant changes in positive affect over time *F*_(1, 198)_ = 25.71, *p <* 0.001, η^2^ = 0.208. Significant effect of attribution of the retrieved memory to one's own past *F*_(1, 98)_ = 4.66, *p* = 0.03, η^2^ = 0.045. Positive memory tasks increased PANAS PA when they were vicarious memories *F*_(1, 98)_ = 6.95, *p* = 0.01, η^2^ = 0.066 but not after the autobiographical retrieval task *F*_(1, 98)_ = 0.548, *p* = 0.461. Performance of participants in the negative group produced a decrease in PANAS PA in the vicarious condition *F*_(1, 98)_ = 10.28, *p* = 0.002, η^2^ = 0.095 while it increased in the autobiographical condition *F*_(1, 98)_ = 88.91, *p <* 0.001, η^2^ = 0.456; significant interactions between time and assignment *F*_(1, 98)_ = 26.56, *p <* 0.001, η^2^ = 0.213, assignment and valence *F*_(1, 98)_ = 37.16, *p <* 0.001, η^2^ = 0.275; and time, valence, assignment *F*_(1, 98)_ = 24.78, *p <* 0.001, η^2^ = 0.202.
			Interpersonal Reactivity Index (IRI) (Davis et al., 1994)		
(Ozawa 2021)	*N =* 72 (37 women and 35 men) Age: 18–30 years	To determine, using a mood induction procedure, how many emotions from six categories (surprise, fear, anger, disgust, sadness, and happiness) participants experience and how these emotions correlate.	Positive and Negative Affect Scale (PANAS) (Watson et al., 1988)	The task began with a relaxation based on deep breathing. Subsequently, participants were asked how unpleasant they felt. Instructions were then given to recall stressful interpersonal events. Guided questions were asked to focus on the event (details and behaviors of other people who appeared in the event). Finally, participants were asked to recall the emotions they experienced at the time of the event.	The items “disgust” (4.43 ± 1.25), “sadness” (4.25 ± 1.45) and “anger” (4.06 ± 1.27) presented the highest scores, followed by “fear” (2.56 ± 1.50) and “surprise” (2.18 ± 1.33); “happiness” (1.32 ± 0.62) scored lowest. A one-way repeated measures analysis showed the main effect of emotion items *F*_(5, 355)_ = 88.76, *p <* 0.001, η^2^ = 0.56. Disgust, sadness, and anger scored higher than fear (*p <* 0.001; *d* = 1.34, 1.15, 1.07), surprise (*p <* 0.001; *d* = 1.74, 1.49, 1.45) and happiness (*p <* 0.001; *d* = 2.87, 2.33, 2.49), and fear and surprise scored higher than happiness (*p <* 0.001; *d* = 0.95, 0.75). To analyze the relationships between emotions, inter-correlations were examined: the item “surprise” correlated positively with the items “fear” (*r* = 0.35, *p* = 0.003), “anger” (*r* = 0.27, *p* = 0.021), “disgust” (*r* = 0.26, *p* = 0.029) and “sadness” (*r* = 0.32, *p* = 0.006). The item “fear” correlated positively with “sadness” (*r* = 0.40, *p <* 0.001), and similarly, the item “anger” with “disgust” (*r* = 0.48, *p <* 0.001). The unpleasant emotion item had significantly higher post-scores compared to pre-scores, *t*_(71)_ = 16.34, *p <* 0.001, *d* = 1.93. The PANAS NA and PA scales showed significant changes in post-scores compared to pre-scores; NA: *t*_(71)_ = 7.59, *p <* 0.001, *d* = 0.90, PA: *t*_(71)_ = −6.53, *p <* 0.001, *d* = 0.77.
(Pacheco-Unguetti and Parmentier 2014)	*N =* 40 (3 men and 37 women) Age: 17–32 years	To examine, in a controlled laboratory task, whether sadness is associated with increased distraction by irrelevant distractors.	Positive and Negative Affect Scale (PANAS) (Watson et al., 1988) Scale for Mood Assessment (EVEA) (Sanz, 2001) Scale for Specific Emotions (SSE) (Pacheco-Unguetti and Parmentier, 2014)	Participants were randomly assigned to two experimental conditions: Sadness condition: generation of the autobiographical memory (saddest memory of their life, eliciting as many details as possible). The event was recalled for 4 min. After this, participants were given 5 min to write it down on paper. During the task, background music was played. Neutral condition: generation of autobiographical recall (recalling a recent trip to a grocery store with as many details as possible). The event was recalled for 4 min. After this, participants were given 5 min to write it down on paper. During the task, background music was played.	For the PANAS PA, main effects were found for Time *F*_(1, 38)_ = 4.70, MSE = 19.19, *p* = 0.036, η^2^ = 0.11; Group *F*_(1, 38)_ = 9.12, MSE = 42.92, *p* = 0.004, η^2^ = 0.19, and Time X Group interaction *F*_(1, 38)_ = 16.46, MSE = 19.19, *p <* 0.001, η^2^ = 0.30. The two groups differed before the induction procedure, but post-induction PA was lower in the sadness group compared with the neutral group *F*_(1, 38)_ = 17.56, MSE = 40.17, *p <* 0.001, η^2^ = 0.31. As regards the PANAS NA, significant effects were found for Group *F*_(1, 38)_ = 5.20, MSE = 28.51, *p* = 0.028, η^2^ = 0.12; Time *F*_(1, 38)_ = 5.58, MSE = 16.16, *p* = 0.023, η^2^ = 0.13, and Group x Time interaction *F*_(1, 38)_ = 20.04, MSE = 16.16, *p <* 0.001, η^2^ = 0.34. The pre-induction scores did not differ between groups, but, post-induction, NA was significantly higher in the sadness group than in the neutral group *F*_(1, 38)_ = 16.74, MSE = 27.21, *p <* 0.001, η^2^ = 0.30. Regarding the scores on the SSE, the sadness emotion subscale revealed main effects for Group, *F*_(1, 38)_ = 13.63, MSE = 6.31, *p <* 0.001, η^2^ = 0.26, and Time, *F*_(1, 38)_ = 44.01, MSE = 3.49, *p <* 0.001, η^2^ = 0.53. The Group x Time interaction was also significant, *F*_(1, 38)_ = 69.01, MSE = 3.49, *p <* 0.001, η^2^ = 0.64, which shows that the sadness group scored significantly lower than the neutral group prior to the induction, *F*_(1, 38)_ = 5.46, MSE = 3.58, *p* = 0.025, η^2^ = 0.12, but scored higher after the procedure, *F*_(1, 38)_ = 49.44, MSE = 6.23, *p <* 0.001, η^2^ = 0.56. For the neutral emotion subscale, the main effect for Group was not significant (*F* < 1), but the main effect for Time was *F*_(1, 38)_ = 18.12, MSE = 6.48, *p <* 0.001, η^2^ = 0.32. The Group x Time interaction was found to be marginally
					significant, *F*_(1, 38)_ = 3.90, MSE = 6.48, *p* = 0.056, η^2^ = 0.09. There were no prelevel differences in the scores between the groups (*F* < 1), although the neutral group reported significantly higher scores than the sadness group in the post-induction measures, *F*_(1, 38)_ = 4.60, MSE = 7.03, *p* = 0.038, η^2^ = 0.10. The scores on the EVEA revealed significant main effects of Group, *F*_(1, 38)_ = 12.89, MSE = 8.14, *p <* 0.001, η^2^ = 0.40. The interaction between these variables was also significant, *F*_(2, 76)_ = 29.04, MSE = 1.74, *p <* 0.001, η^2^ = 0.43. The scores for the two groups were similar before the induction (*F* < 1), but were significantly higher in the sadness group after completing the induction phase *F*_(1, 38)_ = 41.60, MSE = 4.04, *p <* 0.001, η^2^ = 0.52.
(Pacheco-Unguetti and Parmentier 2016)	*N =* 44 (8 men and 36 women) Age: 17–28 years	To examine, in a controlled laboratory task, whether happiness is associated with increased distraction by irrelevant distractors.	Positive and Negative Affect Scale (PANAS) (Watson et al., 1988)	Participants were randomly assigned to two experimental conditions: Happiness condition: generation of the autobiographical memory (happiest memory of their life, eliciting as many details as possible). The event was recalled for 4 min. After this, participants were given 5 min to write it down on paper. Neutral condition: Same procedure as in the previous study (Pacheco-Unguetti and Parmentier, 2014). In both conditions, and during the task, participants were exposed to three background musical pieces previously selected for their ability to induce a positive, negative and neutral mood, respectively.	The interaction between these variables was not statistically significant, *F*_(1, 42)_ = 2.61, MSE = 20.72, *p* = 0.113, η^2^ = 0.05, although, numerically, the data show an increase in positive scores following the positive mood induction. As for the PANAS-NA measure, a significant main effect of time was observed, *F*_(1, 42)_ = 8.71, MSE = 13.03, *p* = 0.005, η^2^ = 0.17. The main effect of group was not significant, *F*_(1, 42)_ = 2.52, MSE = 27.5, *p* = 0.119, η^2^ = 0.05, and neither was the time x group interaction, *F*_(1, 42)_ = 0.22, MSE = 13.03, *p* = 0.639, η^2^ = 0.005.
(Philippot et al. 2003) Study 2	Study 2: *N =* 60 (51 women and 9 men) Age:18–23	To determine whether priming emotional memory processing affects subsequent mood induction when participants are confronted with an external stimulus	Positive and Negative Affect Scale (PANAS) (Watson et al., 1988) Beck Depression Inventory (BDI) (Beck et al., 1988a,b) Negative Mood Regulation Scale (NMRS) (Catanzaro and Mearns, 1990) Differential Emotion Scale (DES) (Izard et al., 1974)	The experiment consisted of two sessions, one based on priming by means of an autobiographical memory and semantic task and the other on watching emotional movie excerpts. In the recall session, 4 trials were performed after a 90-s relaxation period. After brief instructions, the participant was asked to recall two specific personal events and two general events related to the themes of the film excerpt (4 in total) for which the experimenter read to the participants the general theme of these excerpts. The themes were related to injustice (anger), loss (sadness), threat (fear) and meeting again with a loved one (happiness). They were asked to describe the memory orally and rate it in emotional terms. The participants in the semantic priming group (control) were told the themes of the emotional movie excerpts and were then asked to perform a verbal association task. In the second phase (1 week later), participants were briefly reminded of the first general or specific autobiographical memory generated in Phase 1 in order to reactivate this memory for 20 s. This was then done for the second memory and so on. Subsequently, participants were shown the movie excerpts and, after each one were asked to describe again their emotional state.	The authors observed significant effects between the emotional content of the movie clip and the items on the DES *F*_(27, 1539)_ = 59.2, *p <* 0.001. They also observed an effect of the priming condition, which was significant in the case of happiness *F*_(18, 513_) = 2.8, *p <* 0.001 (with no differences between the semantic and specific conditions and higher general scores) and anger *F*_(15, 513)_ = 1.8, *p <* 0.059 (with no differences between the semantic and specific conditions and higher general scores), and no effect for sadness *F*_(18, 513)_ = 1.40, *p* = 0.13 or fear *F*_(18, 513)_ = 1.14, *p* = 0.31. As for the PA and NA measures, a substantial main effect for the emotional movie was observed *F*_(3, 171)_ = 186.7, *p <* 0.001, as well as a significant effect according to the priming condition *F*_(2, 57)_ = 4.82, *p <* 0.012: general memory (*M* = 1.12, *DT* = 0.40), specific memory (*M* = 0.88, *DT* = 0.34) and semantic (*M* = 0.84, *DT* = 0.43). The comparisons showed that the semantic and specific conditions did not differ significantly, but the general condition did differ with respect to the combined mean of the semantic and specific conditions.
(Quigley et al. 2021)	*N =* 118 82% women Age: 17–40	To analyze the effects of adopting a mood orientation on the valence of autobiographical memories following an emotional induction.	Beck Depression Inventory (BDI-II) (Kühner et al., 2007) Positive and Negative Affect Scale (PANAS) (Watson et al., 1988) Single-Item Mood Scale (SIMS)	Participants were individually evaluated by completing the assessment prior to the start of the induction, viewing the video clip. They were then randomly assigned to the defined conditions of rumination or reflection, completing the tasks displayed on the screen. They were then asked to complete a recall task and to re-measure the target variables for purposes of comparison.	Participants' moods were significantly more negative after emotional inductions PANAS *F*_(1, 116)_ = 22.68, *p* = < 0.001 and SIMS *F*_(1, 116)_ = 95.03, *p <* 0.00. Depressive symptomatology was significantly associated with the valence of memories *b* = −0.03, SE = 0.01, *t* = −3.39, *p* = 0.001. Depressive symptoms significantly predicted PANAS scores from induction to recall task *b* = 0.28, SE = 0.04, *t* = 6.21, *p <* 0.001, and SIMS scores *b* = −0.13, SE = 0.02, *t* = −6.85, *p <* 0.001. Participants who recalled more positive memories experienced greater mood repair *b* = −1.30, SE = 0.41, *t* = −3.17, *p* = 0.002. The evocation of positive memories was associated with greater mood repair *b* = 0.91, SE = 0.19, *t* = 4.88, *p <* 0.001.
(Seebauer et al. 2016)	Study 1: *N =* 87 Study 2: *N =* 57	To analyze the impact of positive memory processing and its influence on mood	(BDI-II) (Kühner et al., 2007) 9-point Likert scale for the emotions of sad, happy, bad, pride, love and attachment Spontaneous Use of Imagery Scale (SUIS; Reisberg et al., 2003).	Study 1: The participants undertook a sadness/negative mood induction using two processing modes (high and low concrete) The participants viewed a film clip validated for the induction of negative mood. An additional, more recent movie clip for inducing negative mood was also introduced given the age of the first one. Subsequently, the participants were instructed to recall a positive autobiographical memory with the guidance of the experimenter, introducing a mood repair phase through a differently processed positive memory. They were randomly assigned to one of 3 conditions depending on how they had to think about the generated memory (abstract thinking, low concrete processing, and high concrete processing). Study 2: The procedure followed was similar to that in Study 1. The participants were randomly assigned to one of the conditions designated as social memory (involving important attachment figures) or achievement (e.g., graduation) using only one type of processing, concrete. Once completed, they were re-evaluated to analyze possible changes.	Study 1: The negative mood induction showed a significant effect for Time *F*_(1, 84)_ = 159, *p <* 0.001, η^2^ = 0.65. No significant interaction was found between Condition and Time *F*_(2, 84)_ = 178, *p <* 0.8371, η^2^ = 004. Mood increased significantly during mood repair in all conditions (*p <* 0.001), although the concrete processing condition showed a better mood state *F*_(1, 84)_ = 6.14, *p <* 0.015, η^2^ = 0.068. Mood and self-rated intensity were significantly and positively correlated *r* = 0.37, *p* = 0.004. Study 2: The negative mood induction showed a significant effect for Time a *F*_(1, 55)_ = 162.98, *p <* 0.001, η^2^ = 0.75. There was no significant interaction between Condition and Time *F*_(1, 55)_ = 2.71, *p* = 0.105, η^2^ = 0.47. In the specific emotions, pride scored higher in the achievement condition *F*_(1, 55)_ = 8.97, *p* = 0.004, η^2^ = 0.140, while love scored higher in the social memory condition *F*_(1, 55)_ = 7.35, *p* = 0.009, η^2^ = 0.118.
(Speer and Salgado 2017)	*N =* 134 (90 women and 44 men) Age M = 20.8(4.2)	To analyze whether the evocation of autobiographical memories with a positive content reduces stress response	Positive and Negative Affect Scale (PANAS) (Watson et al., 1988) Beck Depression Inventory (BDI) (Beck et al., 1988a,b) Connor Davidson Resiliency Scale (CD-RISC) (Connor and Davidson, 2003)	Participants were randomly assigned to 4 experimental groups. After an initial measure of mood, they completed an autobiographical recall task (24 positive or 24 neutral), providing details of the event. During each trial, they were required to elaborate on the memory. They were then asked to rate the emotional intensity and feeling of the memory.	A significant interaction between valence and condition is observed for the negative post-recall effect *F*_(1, 130)_ = 5.53, *p* = 0.04. The positive stress group showed lower PANAS NA after recall than the neutral group (positive group *M* = 14.48, *SD* = 4.12, neutral group *M* = 17.06, *SD* = 6.88, *t* = −1.68, *p* = 0.069). Indirect effect of emotion during recall (through resilience) on subsequent mood was significant *B* = 6.53, *SE* = 3.28, *t* = 2.00, *p* = 0.05.
(Xue et al. 2018)	*N =* 49 (27 women and 22 men) Age: 18–26 M = 22.10(2.15)	To examine the influence of induced self-centered mood on music preference.	Chinese version of the Positive and Negative Affect Scale C-PANAS (Qiu et al., 2008; Watson et al., 1988)	Participants were randomly assigned to one of three experimental conditions. Sad mood condition = they were asked to recall three sad autobiographical memories. Happy mood condition = they were asked to recall three happy autobiographical memories. Neutral mood condition = they were asked to write down the names of the last three meals beginning from the latest to the oldest. Before leaving the laboratory, those in the sad mood condition viewed a pleasant 2-min video to release their induced sadness.	Sad mood condition: *F*_(1, 15)_ = 7.88, *p* = 0.013, η^2^ = 0.345. The interaction was significant *F*_(1, 15)_ = 31.24, *p <* 0.001, η^2^ = 0.675. The pre-test score was lower than the score after the positive emotion dimension test (*p <* 0.001) and higher in the negative emotion dimension (*p* = 0.001). Happy mood condition: *F*_(1, 15)_ = 44.59, *p <* 0.001, η^2^ = 0.748. The interaction was also significant, *F*_(1, 15)_ = 13.31, *p* = 0.002, η^2^ = 0.470. The pre-test score was higher than the score following the positive emotion dimension (*p* = 0.021) and lower in the negative emotion dimension (*p* = 0.037). Neutral mood condition: *F*_(1, 16)_ = 27.10, *p <* 0.001, η^2^ = 0.629. The interaction was not significant, *F*_(1, 16)_ = 2.21, *p* = 0.157, η^2^ = 0.121.

### 2.5 Data analysis

A meta-analysis was performed with the publications selected for the systematic review and that also had numerical data on pre- and post- mood induction results using the PANAS scale (Watson et al., 1988).

After extracting the pre- and post-test means and standard deviations for the PANAS negative affect (NA) and positive affect (PA) subscales, we calculated the effect sizes using standardized pretest-posttest mean change index with Hedges' correction for small sample sizes. In the case of the study by (Monno et al. 2024), the table reports only statistically significant differences. To avoid bias from unreported non-significant contrasts, we imputed an effect size of 0 for those cases. Attempts to obtain the missing statistics from the authors were unsuccessful. The analyses for overall effect size were performed using the random-effects method, assuming a variation between effect sizes across studies. The Q test for homogeneity and the I2 statistic for heterogeneity were used to assess heterogeneity in variance between effect sizes across studies. Egger's test was used for the analyses of publication bias. To classify the effect sizes, Cohen's criterion was used (0.20–0.49—small effect; 0.50–0.79 medium effect; ≥0.80- large effect). Taking the heterogeneity analyses into account, we evaluated the impact of possible moderators on the results by means of subgroup analyses according to the type of stimulus used in the induction (verbal, images or music), whether the method included a prior negative emotion induction, or whether the process of retrieving the AMs involved writing them down. It was considered inappropriate to perform a meta-regression with a combination of several moderators due to limited number of studies.

We classified an induction as positive when instructions or materials targeted positive autobiographical recall, and as neutral when they targeted neutral recall or materials. It was classified as negative when they focused on an induction associated with negative memories. For both types, we extracted the pre- and post-change scores in PANAS PA and NA. We then conducted planned subgroup comparisons based on cue modality, presence of a prior negative induction, and task format.

## 3 Results

### 3.1 Search and data extraction results

The bibliographic references of the studies included in the present review are listed in reference section, while Table 1 provides specific details on each article. Below, we describe the results of these studies, classified according to the type of stimulus used to access autobiographical memory, and categorized by unimodal and bimodal procedures (Table 1).

### 3.2 Unimodal procedures

#### 3.2.1 Verbal

The study by (Allen et al. 2014) implemented two experiments in which participants recalled and described AMs with different emotional valence. In the first, the negative and neutral valences of the memory were manipulated, finding that the negative condition induced negative emotional states, particularly sadness. In the second, the positive valence was added to the previous ones, with the authors finding that the positive condition activated positive emotions (e.g., joy, affection), while the negative condition generated negative emotions (e.g., sadness, anger).

In the study by (Burns et al. 2003), the participants were asked to recall and describe aloud a recent event that evoked the emotion corresponding to their assigned condition (anger, sadness, or joy). The results showed that, following the MIP, PA scores increased for the joy condition and decreased for the sadness condition. No significant changes in PA were observed for the anger condition. As for the NA scores, these increased for the sadness and anger conditions but decreased for the joy condition.

In their study, (Fabiansson et al. 2012) asked participants to recall an anger-inducing event that had occurred within the past 12 months. They then engaged in three emotion regulation conditions: reappraisal, analytical rumination, and anger-focused rumination. Reappraisal induced higher levels of anger than the other two conditions. Additionally, self-reported levels of anger remained constant across conditions throughout the task.

The study by (Gendolla et al. 2001) randomly assigned participants to four experimental conditions: negative music, positive music, negative AM and positive AM. The task consisted of recalling a life event according to the assigned condition and describing it vividly in writing. The results showed that both music and memories (positive and negative) significantly affected the participants' mood.

In the study by (Gendolla et al. 2005), participants were instructed to recall and describe a positive or negative life event in a vivid and emotional manner. They were also subjected to a self-focus (participants sat in front of a mirror) or non-self-focus (participants remained facing a wall) manipulation. The results showed successful manipulation for both positive and negative mood, with no differences found according to the self-focus/no self-focus condition.

In their study, (Jallais and Gilet 2010) randomly assigned the participants to eight experimental conditions, which included four emotional states (happy, serene, angry, and sad) and two procedures (AM vs. combined music and guided imagery procedure). Participants were asked to recall and write down a memory or undergo music-guided imagery according to their condition. Although there were no significant differences in emotional valence between the two procedures, the AM generated a greater change in sadness compared to the combined procedure. In terms of arousal, the AM procedure generally produced a greater increase, although the combined procedure yielded a more robust increase in serenity and happiness. There were no differences in arousal for the sad and angry conditions.

In the study by (Laco et al. 2021), participants were randomly assigned to three experimental conditions (happy AM, neutral AM, and happy music). In the recall task, participants were required to imagine a past event as vividly as possible, assisted by a series of guiding questions. The participants in the happy AM group were more positive than those in the neutral AM group and the music condition group. Furthermore, the group in the musical condition was significantly more enthusiastic than the happy and neutral AM groups.

In the study by (Lievaart et al. 2017), the participants were asked to write down a detailed account of three events in which they had become very angry with someone else. Then the experimenter selected the least solved event for participants to attempt to relive during a subsequent interview. The results highlighted a significant change in the emotion of anger, with participants experiencing increased anger after the MIP.

The participants in the study by (MacKinnon et al. 2013) prepared two written autobiographical scripts describing their most recent moment of sadness and happiness, with the aim of visualizing and experiencing each emotion. The participants reported a greater positive compared to negative emotion during the happiness condition, and a greater negative compared to positive emotion during the sadness condition.

In the study by (Nourkova and Gofman 2022), the participants performed a direct AM task (a description as detailed as possible of past personal events) and an indirect one (description of a past event on behalf of a favorite fictional characters) and under the happy and unhappy condition. The happy AM tasks resulted in higher PA scores, with these being slightly higher in the direct recall task. Under the unhappy recall conditions, there was a slight decrease in PA in indirect recall and an increase in direct recall. NA scores were lower after both direct and indirect happy recall tasks. Additionally, direct unhappy recall inhibited NA, while no significant effects were found for indirect unhappy recall.

In the study by (Ozawa 2021), the authors asked the participants to recall stressful events in daily life and then focus on a specific incident with the guidance of the experimenter. The participants reported that the emotions of disgust, sadness, and anger were felt most prominently, followed by fear and surprise, while happiness was scarcely felt. Pre- and post-induction PANAS scores showed emotional changes, especially in unpleasant emotions. Increased NA and decreased PA were associated with increased sadness and fear.

In the study by (Quigley et al. 2021), participants watched a film clip to induce a sad mood. Subsequently, they were randomly assigned to two conditions: reflection or rumination, in which they focused on their feelings. They were then asked to vividly recall and write down five specific AMs from their high school years. Mood was measured at the beginning of the experiment, after the negative induction, and after the recall task. It was found that participants whose AMs were more positive experienced greater mood repair after the negative induction.

The study by (Seebauer et al. 2016) involved two experiments. In the first, after inducing a negative mood (using video clips), participants performed a mood repair task using positive AMs in three memory processing conditions: abstract, low concrete and high concrete. The three conditions had a mood repair effect, with substantive increases in the case of generating social memories (e.g., trips, nature). Subjective imagery intensity was crucial in enhancing mood. In the second experiment, after inducing negative emotions, the participants were randomly assigned to AM conditions of social or achievement content. These findings suggest the possibility of inducing specific positive emotions by varying the memory content.

In their study, (Speer and Salgado 2017) presented the participants, in a first session, with 84 common life event cues (e.g., vacations). From these, they chose, based on their association with AMs, the 24 that were most positive and the 24 that were most neutral. The cues were then used in a second session for participants to generate AMs. The results showed that positive recall was associated with lower NA compared to neutral recall. There were no differences between positive and neutral memory with respect to PA, although the more positive the memories were, the more enhanced was the participants' mood.

The study by (Xue et al. 2018) induced a sad, happy, or neutral mood in participants by asking them to recall three autobiographical events corresponding to the valence of the condition they were assigned to. The results showed an increase in negative emotions and a decrease in positive emotions in the sad condition, while an increase in positive emotions and a decrease in negative emotions were observed in the happy condition.

In the study by (Monno et al. 2024), participants were required to listen to audio clips and choose the mood they experienced for at least 1 min. After this, the task began, during which 60 sentences were shown to each participant to be associated with an AM. The results showed that it had immediate effects on the participants' emotions. The positive, neutral, and negative emotional responses indicated that stimulus selection and individual characteristics can influence the outcomes of mood induction.

#### 3.2.2 Musical

In the study by (Barrett and Janata 2016), the participants performed an AM task based on listening to 360 random musical excerpts from songs released when they were aged between 7 and 19 years. The results showed that 59% of the excerpts were rated as being weakly nostalgic and 40% as moderately nostalgic. The ratings of nostalgia were highly correlated with the autobiographical salience scores.

The work by (López-Cano et al. 2020) comprised two studies. In the first one, songs from the pop charts were used as autobiographical stimuli. The music was found to generate an increase in PA and a decrease in NA, especially in older adults. Regarding the effect of AM recall on positive mood, the results pointed to a slight and significant increase. The second study used excerpts of songs taken from the charts, divided into native vs. international popular music. The results showed a significant effect of the MIP, enhancing PA and reducing NA. Better results were achieved with native songs and with those from the reminiscence bump period (preference for recalling events that occurred in adolescence and early adulthood).

### 3.3 Visual

The study by (Carretero et al. 2020) used two types of images as autobiographical stimuli: images from the International Affective Picture System IAPS (Lang et al., 2008) and participants' personal photographs. After a negative emotion induction procedure using a film clip, participants retrieved positive AMs when viewing the images. The results showed that, regardless of the type of image, the task significantly improved participants‘ mood, reducing arousal and enhancing pleasantness. Despite the impersonal nature of the IAPS, similar levels of nostalgia and reliving of memory were reported compared to personal photographs. In the IAPS condition, mood repair depended on the feeling of reliving the memory to a greater extent than in the personal picture condition.

The study by (Fernández-Pérez et al. 2023) implemented three experimental conditions according to the type of image used, namely, standardized (IAPS; Lang et al., 2008), images of places (taken from the Internet) and personal photographs, and two age groups (young and old). Following a negative mood induction using a movie clip, participants retrieved specific positive MAs associated with the previously selected images (depending on the experimental condition). All three groups experienced an increase in PA and a decrease in NA. Personal photographs were particularly effective in reducing NA, followed by those associated with places and IAPS images. Additionally, the greater effectiveness of the personal images was highlighted, given their greater personal relevance, the ability to re-experience the event, and higher levels of positivity and nostalgia in the memories generated.

In the second experiment from the study by (Philippot et al. 2003), the participants were asked to generate specific and general AMs related to film clips that evoked anger, sadness, happiness and fear. Each excerpt activated a specific pattern of emotions, showing a significant association with the scores on the Differential Emotion Scale (DES; Izard et al., 1974), based on a list of emotional adjectives. The results showed more intense emotions for the general memory condition compared to the specific memory condition.

### 3.4 Bimodal and multimodal procedures

#### 3.4.1 Verbal and musical

In the study by (Benau and Atchley 2020), participants listened to a piece of music twice. They were asked to think about a sad memory while listening and then to write it down in detail. Following the induction, a significant increase in sadness and fear was observed, as well as a decrease in happiness. Anger levels remained constant. The change induced was more notable for happiness and sadness than for fear and anger. In addition, the memories generated showed components of various emotions, highlighting the difficulty of experiencing primary emotions in isolation when recalling complex events.

The study by (Pacheco-Unguetti and Parmentier 2014) randomly assigned participants to two experimental conditions: generation of sad or neutral AMs that combined listening to musical pieces (chosen for their propensity to induce sadness) and the generation and writing of a memory. The sad group mainly recalled events such as the death of a loved one, loss of a pet and divorce, whereas the neutral group recalled neutral memories. Following the induction, the sad group exhibited lower PA and higher NA, and higher scores on sadness, anxiety, disgust, fear, and anger, and lower scores on happiness and surprise, compared to the neutral group.

In the work by (Pacheco-Unguetti and Parmentier 2016), the participants were randomly assigned to one of two experimental induction conditions, positive or neutral mood, using a combination of background music (selected for its propensity to induce positive mood) and retrieval and writing down of AMs. Following the MIP, PA scores were higher in the positive mood group, albeit with no statistically significant differences with the neutral mood induction group. The NA scores were lower after positive induction, but again there were no statistically significant differences with respect to the neutral induction group.

#### 3.4.2 Verbal and visual

The study by (Dehghani et al. 2022) first asked participants to write down a number of autobiographical happy memories. Subsequently, in guided interviews, participants were asked to retrieve the memories with the help of general pictures matching the content of those memories. The recall was accompanied by a neurofeedback task with two conditions: an experimental group in which the neurofeedback task consisted of increasing or maintaining the height of a bar based on brain activity, and a control group where the signal was randomly generated. The results showed significant changes in both groups in terms of decreased negative mood, increased positive mood, and decreased anxiety.

In the study by (Gotz et al. 2022), participants performed four AM tasks using methods based on virtual reality (VR): (1) talking to a virtual agent; (2) talking to an avatar as the researcher's proxy; (3) thinking quietly about a past experience; and (4) writing or drawing about a past event using a VR pen. In each condition, participants were required to generate a detailed memory for each emotion (sadness, happiness, anger and fear). The results showed the efficacy of the four VR methods of AM in inducing the target emotions. There were no significant differences between the different VR methods in valence, arousal, or dominance. However, the silent-thinking method induced lower arousal and the proxy avatar method induced lower dominance.

#### 3.4.3 Musical, visual and verbal

As autobiographical stimuli, the study by (Cady et al. 2008) used songs from five different eras of the participants' lives: early childhood, grade school, middle school, high school, and college. The participants associated a memory with each era according to the experimental condition: presentation of song title, listening to part of the song, viewing written lyrics of the song, and the album cover. Although PA did not vary significantly in any of the conditions, NA decreased significantly in the auditory and visual image conditions. No differences were found in the emotional intensity experienced between conditions.

### 3.5 Meta-analysis

Finally, 9 studies were included in the meta-analysis, in which 21 different emotional inductions were performed (Cady et al., 2008; Dehghani et al., 2022; Fernández-Pérez et al., 2023; López-Cano et al., 2020; Monno et al., 2024; Pacheco-Unguetti and Parmentier, 2014, 2016; Quigley et al., 2021). All of these studies evaluated the effect of mood induction by autobiographical stimuli, alone or in combination with other methods, using stimuli classified as neutral (10 experiments), positive (8 experiments) or negative (3 experiments). As dependent variables, the overall PANAS negative and positive affect scores were considered. Twenty one sizes for NA and 19 for PA were extracted. A total of 797 individuals with mean ages ranging from 19 to 71 years participated in these studies.

The results were separated according to the valence of the stimulus used in the mood induction. It is worth noting that a negative sign in the results indicates an increase from pre- to post-test, while a positive sign denotes a decrease.

We performed statistical analyses to test for the influence of possible moderators.

#### 3.5.1 Overall effect size. Meta-analysis on the effect of mood induction using neutral autobiographical stimuli

**Figure 2:**
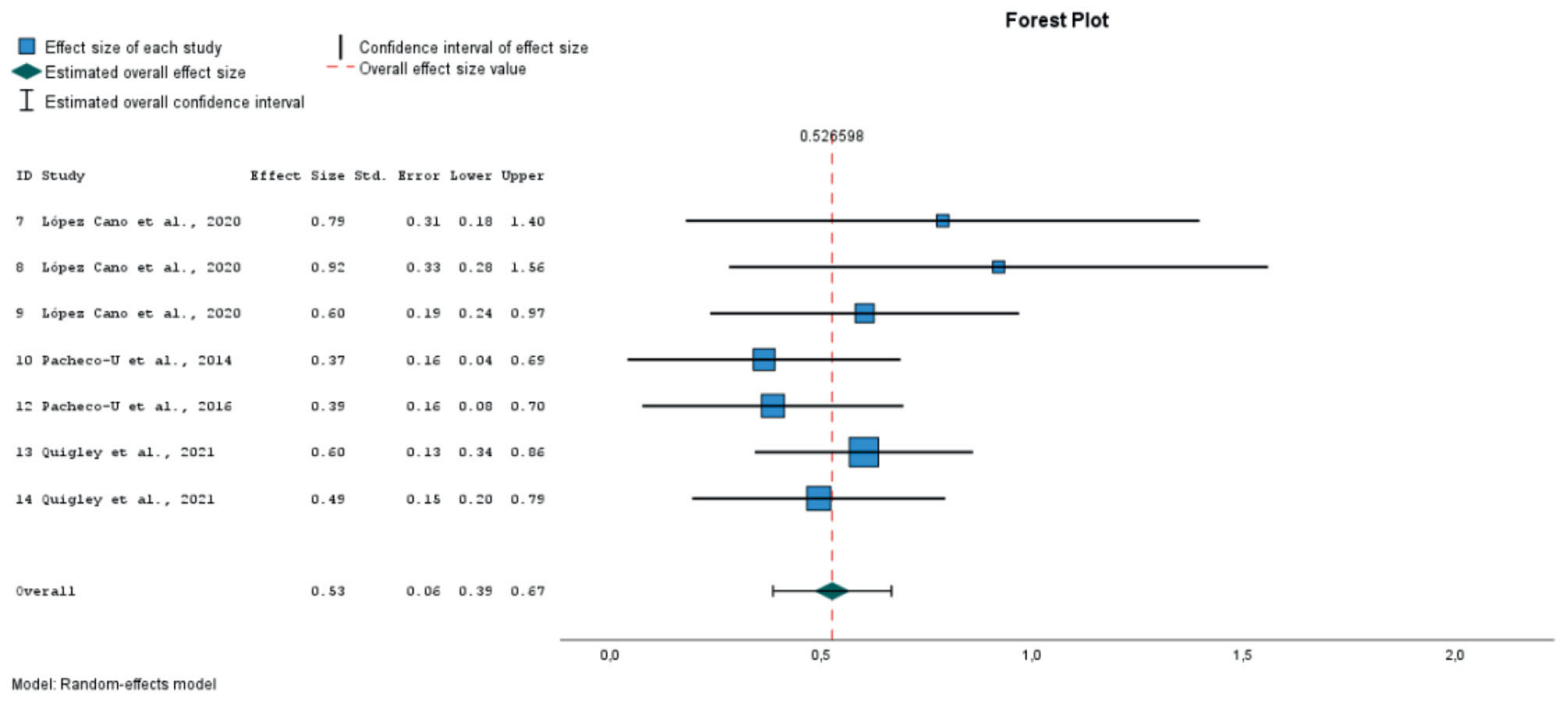
Effect size, overall effect size, and confidence interval using NA stimuli.

For the NA variable, the overall effect size of ten mood induction procedures with neutral autobiographical stimuli was g = 0.43 (SE: 0.08), 95% CI (0.24; −0.61) with a statistically non-significant result in the homogeneity Q-test [Q(9) = 15.80, *p* = 0.071] and a heterogeneity measure, I2, of 39.2. Publication bias, as measured by Egger's test, was not statistically significant (*p* = 0.448). Figure 2 shows the effect sizes for each study, together with the overall effect size, as well as the confidence intervals.

**Figure 3:**
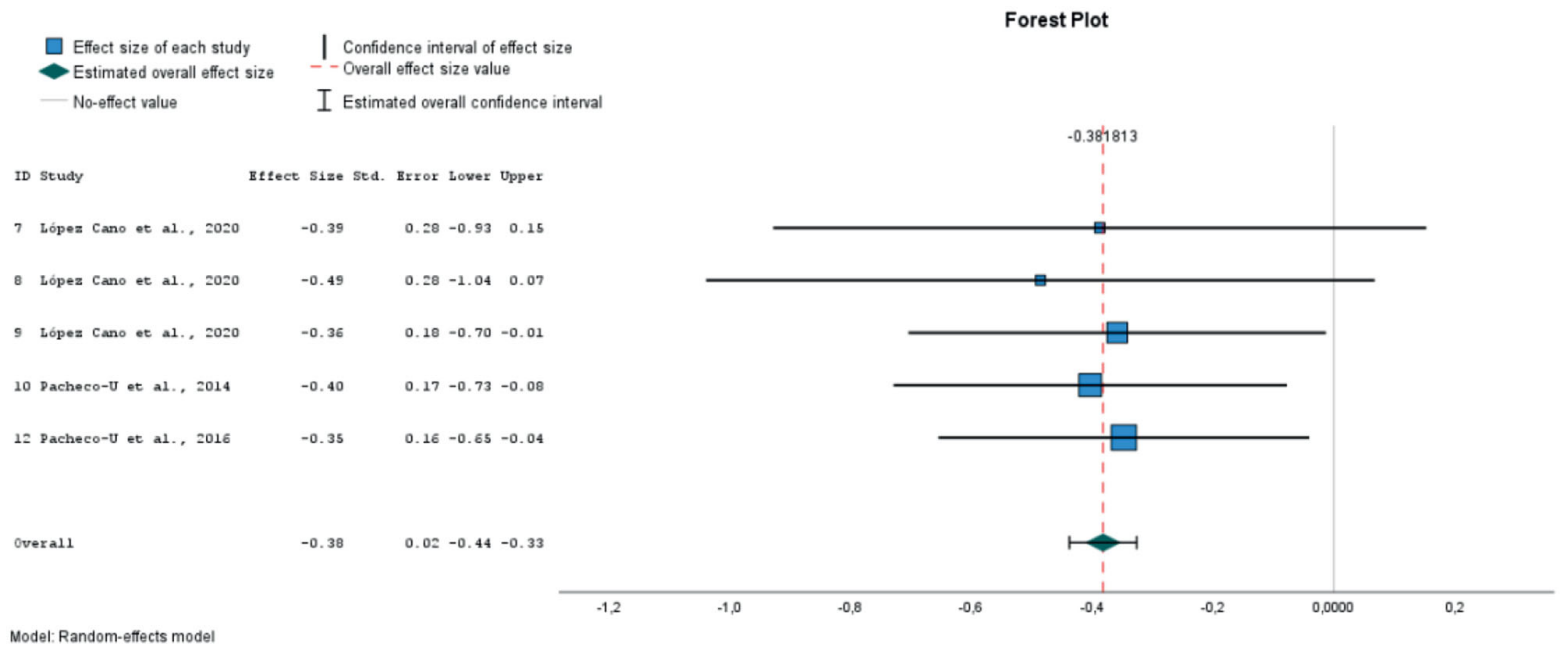
Effect size, overall effect size, and confidence interval using PA-NA stimuli.

For the PA variable, the overall effect size for eight mood induction procedures with neutral autobiographical stimuli was g = 0.10 (SE:0.25) 95% CI (−1.61; 1.81) with a statistically significant result in the Q-test of homogeneity [Q(7) = 66.65, *p* = < 0.001] and with a heterogeneity measure of 90.7. No statistical significance was obtained in the publication bias analysis (*p* = 0.494). The effect sizes for each study, the overall effect size, and the confidence intervals can be seen in Figure 3.

The effect sizes of induction with neutral autobiographical stimuli are of small practical significance for NA and no practical significance for PA, according to Cohen's (1988) recommendations for typified mean difference, with a reduction in NA at posttest compared to pretest.

#### 3.5.2 Overall effect size. Meta-analysis on the effect of mood induction using positive autobiographical stimuli

**Figure 4:**
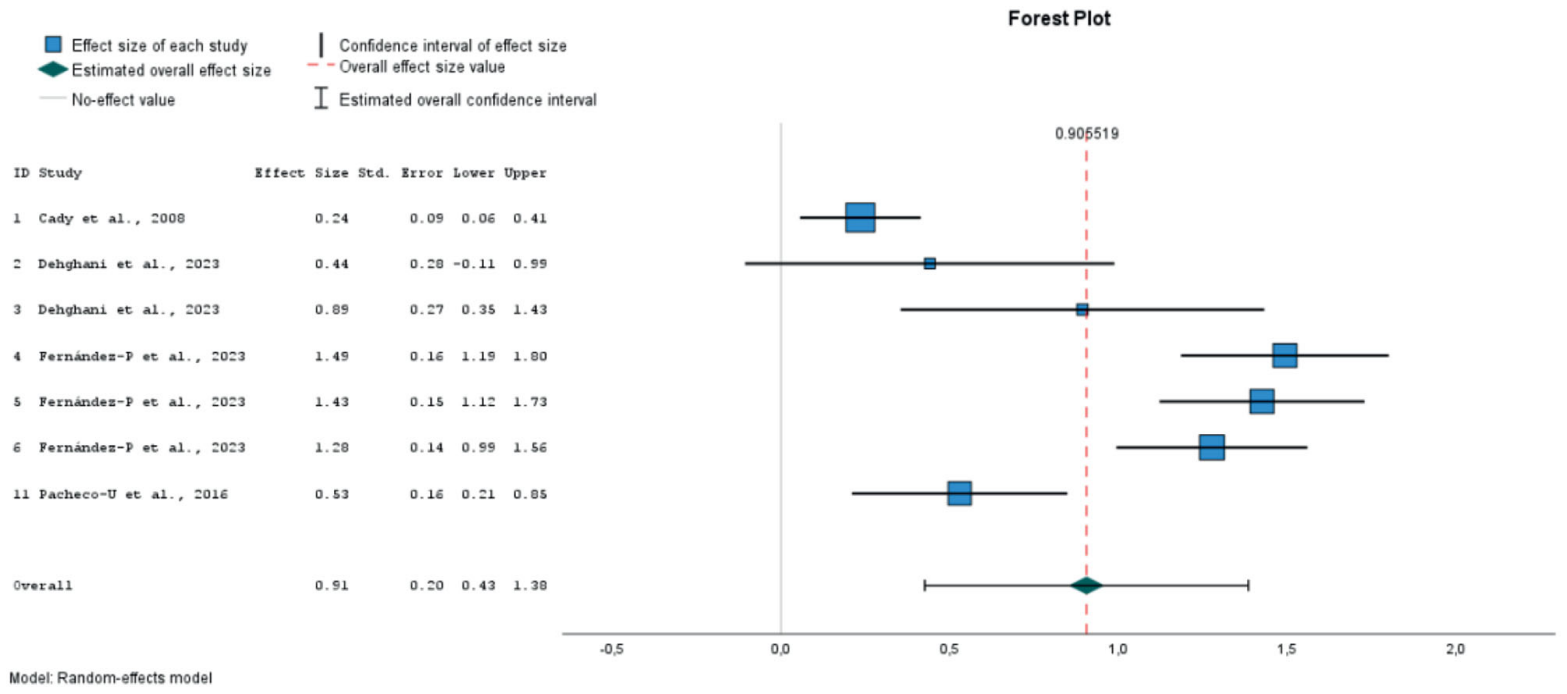
Effect size, overall effect size, and confidence interval using NA-PA stimuli.

For the NA variable, the overall effect size of the eight mood induction procedures using positive autobiographical stimuli was g = 0.92 (SE: 0.17), 95% CI (0.51; 1.32) with a statistically significant result in the Q test of homogeneity [Q(7) = 90.20, *p* < 0.001] and a measure of heterogeneity, I2, of 88.8%. No statistical significance was found in the publication bias analysis (*p* = 0.969). Figure 4 shows the effect sizes for each study, the overall effect size, and the confidence intervals.

**Figure 5:**
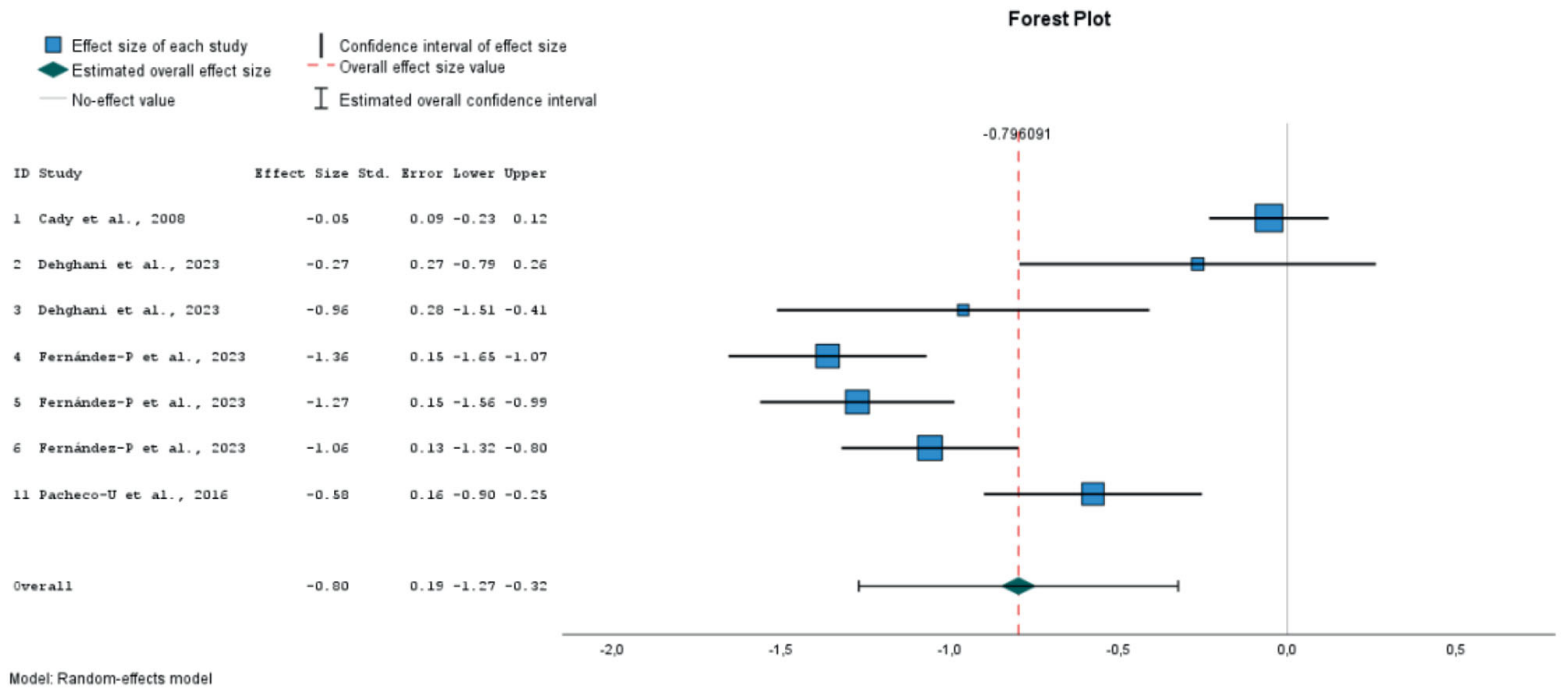
Effect size, overall effect size, and confidence interval using PA-PA stimuli.

For the PA variable, the overall effect size of the eight mood induction procedures using positive autobiographical stimuli was g = −0.70 (SE:0.19) 95% CI (−1.16; −0.24) with a statistically significant result in the Q test of homogeneity [Q(7) = 107.29, *p* < 0.001], and a heterogeneity measure, I2, of 91.9%, with no statistical significance being found in the publication bias analysis (*p* = 0.848). The effect sizes for each study, the overall effect size, and the confidence intervals are shown in Figure 5.

The effect sizes of the induction procedures using positive autobiographical stimuli, are of high practical significance for NA and of medium significance for PA (Cohen, 1988), with NA decreasing and PA increasing from pre- to post-test (Cohen, 1988).

#### 3.5.3 Overall effect size. Meta-analysis on the effect of mood induction using negative autobiographical stimuli

**Figure 6:**
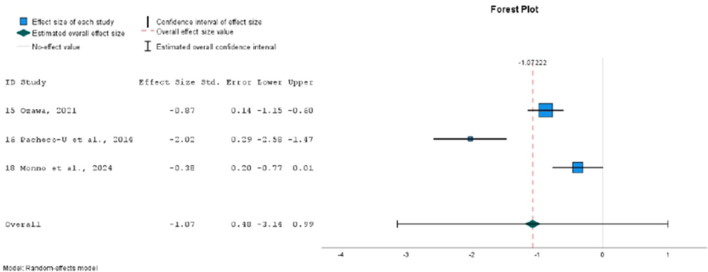
Effect size, overall effect size, and confidence interval using negative stimuli (negative affect).

For the NA variable, the overall effect size of the three mood induction procedures using negative autobiographical stimuli was g = −1.07 (SE: 0.48), 95% CI (−3.14; 0.993) with a statistically significant result in the Q test of homogeneity [Q(2) = 22.38, *p* < 0.001] and a measure of heterogeneity, I2, of 94.1%. No statistical significance was found in the publication bias analysis (*p* = 0.463). Figure 6 shows the effect sizes for each study, the overall effect size, and the confidence intervals.

**Figure 7:**
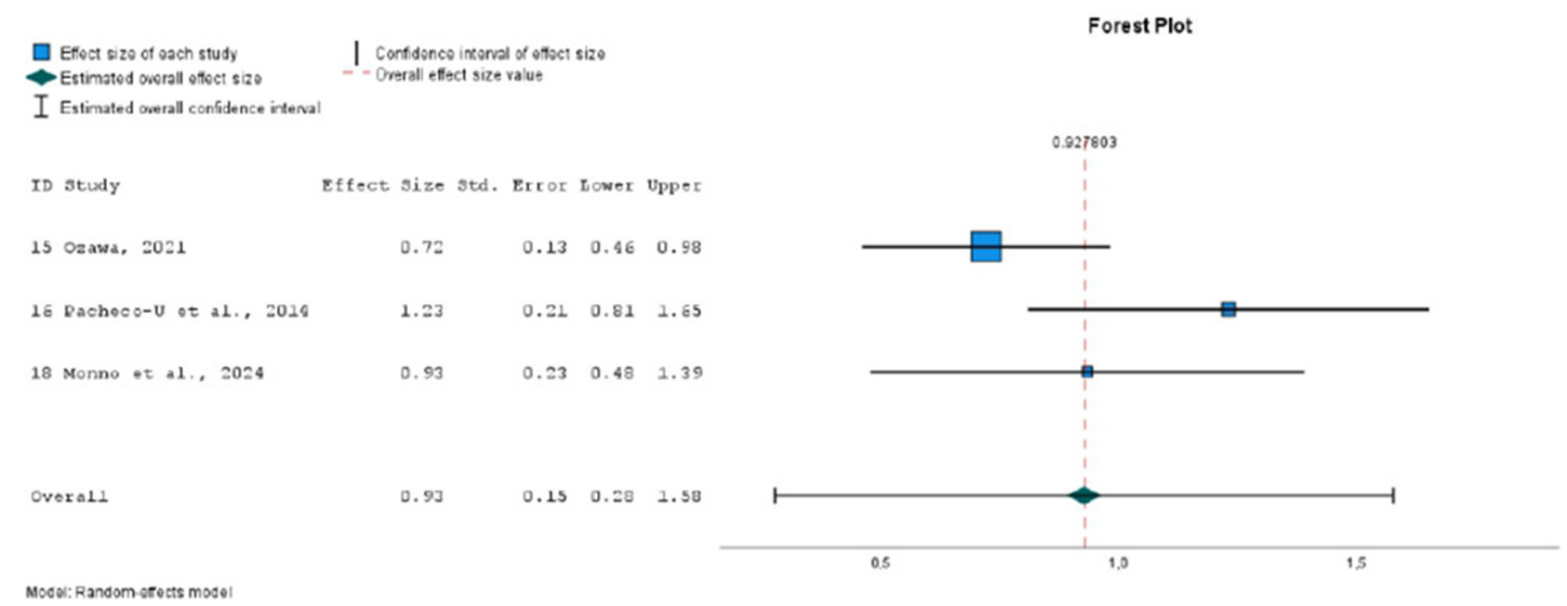
Effect size, overall effect size, and confidence interval using positive stimuli (positive affect).

For the PA variable, the overall effect size of the three mood induction procedures using negative autobiographical stimuli was g = 0.93 (SE:0.15) 95% CI (0.28; 1.56) with a not statistically significant result in the Q test of homogeneity [Q(2) = 4.14, *p* = 0.126], and a heterogeneity measure, I2, of 51.6%, with no statistical significance being found in the publication bias analysis (*p* = 0.422). The effect sizes for each study, the overall effect size, and the confidence intervals are shown in Figure 7.

The effect sizes of the induction procedures using negative autobiographical stimuli, are of high practical significance for both NA and PA (Cohen, 1988), with NA increasing and PA decreasing from pre- to post-test (Cohen, 1988).

#### 3.5.4 Analysis of the moderators in the mood induction procedures using autobiographical stimuli

In view of the heterogeneity detected in the studies using in this meta-analysis, we performed statistical analyses to test for the influence of three possible moderators (the type of stimulus used, classified as verbal cue, images or music, the use of a negative mood induction task before the induction by AM and whether or not the retrieval of autobiographical memories involved writing them down). The results of the induction procedures using neutral and positive stimulus can be found in Tables 2, 3. This procedure was not performed with negative stimulus induction due to the limited number of studies available.

**Table 2:** Analysis of the moderators in the mood induction procedures using neutral autobiographical stimuli.

	**Type of stimulus**					**Negative mood induction task before the mood induction by AM**					**To write the AM**				

	**Verbal cue**		**Music**			**No**		**Yes**			**No**		**Yes**		
	***g (SE)***	**95%** ***CI***	***g (SE)***	***95% CI***	**p** ^*^	***g (SE)***	**95%** ***CI***	***g (SE)***	***95% CI***	**p** ^*^	***g (SE)***	**95%** ***CI***	***g (SE)***	***95% CI***	**p** ^*^
**Variable**															
Negative affect	0.56 (0.10)	0.36: 0.75	0.50 (0.09)	00.33: 0.68	0.694	0.39 (0.10)	0.20: 0.58	0.56 (0.10)	0.36: 0.75	0.226	0.42 (0.12)	0.18: 0.66	0.47 (0.09)	0.30: 0.64	0.727
Positive affect^**^			−0.38 (0.09)	−0.55: −0.21		0.1 (0.24)	0.38: 0.58				0.27 (0.30)	−0.33: 0.864	−0.37 (0.11)	−0.60: −0.15	0.047

AM, autobiographical memory.

^*^p: test of subgroup homogeneity (QB).

^**^There is not data of any study with verbal cue or with negative mood induction task before the mood induction by AM with positive affect measurement.

**Table 3:** Analysis of the moderators in the mood induction procedures using positive autobiographical stimuli.

	**Type of stimulus**					**Negative mood induction task before the mood induction by AM**					**To write the AM**				

	**Images**		**Music**			**No**		**Yes**			**No**		**Yes**		
	***g (SE)***	**95%** ***CI***	***g (SE)***	***95% CI***	**p** ^*^	***g (SE)***	**95%** ***CI***	***g (SE)***	***95% CI***	**p** ^*^	***g (SE)***	**95%** ***CI***	***g (SE)***	***95% CI***	**p** ^*^
**Variable**															
Negative affect	1.16 (0.18)	0.81: 1.51	0.35 (0.14)	0.07: 0.63	< 0.001	0.57 (0.15)	0.27: 0.86	1.39 (0.09)	1.22: 1.56	< 0.001	1.35 (0.08)	1.18: 1.51	0.46 (0.14)	0.19: 0.73	< 0.001
Positive affect	−1.03 (0.17)	−1.37: −0.69	−0.30 (0.26)	−0.81: 0.21	0.019	−0.34 (0.17)	−0.66: −0.01	−1.22 (0.09)	−1.40: −1.04	< 0.001	−0.94 (0.31)	−1.54: −0.34	−0.42 (0.19)	−0.80: −0.04	0.157

AM, autobiographical memory.

^*^p: test of subgroup homogeneity (QB).

Starting with induction using neutral stimuli, there were 10 effect sizes available, of which 5 used music as a stimulus and 2 used verbal cues [the three experiments with neutral induction from the study by (Monno et al. 2024) had to be excluded from this analysis because they included both music and verbal cues in the induction]; 2 used negative emotional induction prior to induction using autobiographical memory and 3 included writing autobiographical memories in their procedure. It was not possible to perform all the calculations on the positive affect variable due to its absence in some studies. The detailed results can be found in Table 2, where it can be seen that the only statistically significant difference is in the positive affect variable between those who did not write down their autobiographical memory and those who did, decreasing in the former case and increasing in the latter.

With regard to induction through positive stimuli, eight effect sizes were available, of which two used music as a stimulus and five used images [the experiment with positive induction from the study by (Monno et al. 2024) had to be excluded from this analysis because they included both music and verbal cues in the induction]; three used negative emotional induction prior to induction through autobiographical memory, and four included writing autobiographical memories in their procedure. The results of this analysis can be found in Table 3. All comparisons were statistically significant, except in the case of the positive affect variable depending on whether the autobiographical memory had been written down.

## 4 Discussion

The aim of the present work was to analyze the effectiveness of MIPs that use autobiographical stimuli, and, consequently, to enhance the understanding of the role of such procedures in the regulation of affective states.

Broadly speaking, in this type of MIP, the participant's mood is assessed at the beginning and end of the experimental task by means of a self-report test, with the PANAS being the most used tool for this purpose (Watson et al., 1988). As a general instruction, participants are typically asked to recall an event in their lives in which they felt a certain emotion, focusing on all possible details of that event (e.g., environment and situation, people present, etc.) and seeking to relive the past experience, letting themselves be carried away by the thoughts and emotions they felt at the time. Some studies encourage participants to relax at the start of the experimental task, establishing a period of habituation and emotional baseline, using various techniques, such as reading popular magazines (Gendolla et al., 2001), listening to relaxing music or doing breathing exercises (López-Cano et al., 2020). On occasions, access to the autobiographical memory is accompanied by a semi-structured interview to help the individual to focus on the specific details of the event in a vivid way, following recommendations from previous studies, such as that by (Watkins and Moberly 2009). The time given to the participant to generate their autobiographical memory can vary from 1 min (Laco et al., 2021) to five (Lievaart et al., 2017), and up to 10 (Jallais and Gilet, 2010).

In our dataset, neutral autobiographical inductions produced only a small decrease in NA and virtually no change in PA, whereas positive inductions yielded a large decrease in NA together with a moderate increase in PA. The apparent advantage of positive over neutral stimuli is likely driven by design features that amplify affective re-experiencing, such as the use of vivid, personally relevant cues (e.g., personal pictures), the delivery of positive recall after a prior negative induction (mood repair), and task-format differences, rather than by the label *per se*. Because “neutral” encompasses heterogeneous procedures, we interpret between-condition differences cautiously and in relation to these methodological variables. Consistent with expectations, negative autobiographical inductions were associated with increases in NA and decreases in PA; however, given the small number of studies and high heterogeneity for NA, these effects should be interpreted with caution.

Regarding potential moderators, our analyses showed different patterns for neutral and positive autobiographical inductions. For neutral procedures, the only significant moderator effect emerged for PA. Participants who wrote their AMs showed an increase in PA, whereas those who did not write showed a decrease; no effects were observed for stimulus modality or for the use of a prior negative induction. In contrast, for positive procedures, most moderator comparisons were statistically significant except for PA in the write vs. no-write contrast, which showed no difference.

Our findings show that most studies focus on the use of cues of a single sensory modality, with few studies employing a combination of two or more modalities. Nevertheless, individuals are exposed on a daily basis to multiple sensory inputs, which simultaneously originate from several modalities, and thus MIPs using more than one sensory modality should allow faster and easier access to AM content (Willander et al., 2015). However, results in this sense are inconclusive. In any event, it is important to understand the properties of the different cues that can be harnessed in memory retrieval, as these will affect the availability of the AM at the moment of recall.

Certain studies have suggested that verbal stimuli, such as words or semantic cues, are not particularly powerful as a means of access to AM content, as they do not adequately represent the sensory information of past events (Willander et al., 2015). The present review shows, however, that such cues tend to be the most commonly used. The techniques used include semi-structured interviews, oral descriptions (Burns et al., 2003; Laco et al., 2021) keywords (Fabiansson et al., 2012) and writing (Jallais and Gilet, 2010; Lievaart et al., 2017).

Nevertheless, our results underline that MIPs that access AM by means of verbal cues are effective in inducing emotions such as sadness and joy or happiness (Nourkova and Gofman, 2022; Xue et al., 2018), anger (Fabiansson et al., 2012; Lievaart et al., 2017), and disgust, fear and surprise (Ozawa, 2021). They also exhibit a similar level of effectiveness to the use of musical cues in inducing positive and negative mood (Gendolla et al., 2001), yield better results than guided imagination in inducing sadness, and similar results with respect to guided imagery in inducing happiness, serenity and anger, although achieving greater emotional intensity (Jallais and Gilet, 2010), and greater positive mood and enthusiasm compared to guided imagination and music (Laco et al., 2021). Additionally, including a neutral state, alongside positive and negative states, yields more notable results compared to the positive and negative conditions alone (Monno et al., 2024).

Additionally, the studies included in this review deploy emotional keywords, as these are considered to facilitate easier access to AM and the emotions associated with it, compared to neutral words (MacKinnon et al., 2013). However, the results of our meta-analysis demonstrate a smaller effect of procedures that include an autobiographical memory writing task (Pacheco-Unguetti and Parmentier, 2016) compared to others that do not (Fernández-Pérez et al., 2023), which runs counter to previous findings pointing to enhanced reliving of the past event if the memory is written down (Mills and D'Mello, 2014).

Musical stimuli are also used with great frequency, with music being a powerful resource that can vividly transport us back in time to past events (Belfi et al., 2016), influencing our emotional responses. This may be due to our listening to music in our daily lives (Greasley and Lamont, 2011), and because, in many cultures, music is traditionally played when celebrating important events (Merriam, 1964). Additionally, AM may be easily accessed through music due to the regularity with which we listen to it, with people having favorite songs that they listen to more frequently (Janssen et al., 2007). These MIPs typically ask participants to listen to musical excerpts, and to then describe any memories the music brings to mind (matching in emotional valence). The excerpts tend to come from songs that have been chart hits and from different life stages (childhood, adolescence, etc.), as well as specific age ranges (e.g., extended childhood from age 7 to 19). There also seems to be a preference for the use of excerpts of popular music rather than classical music, because of the greater familiarity of the former and the fact that popular music is experienced in everyday situations, thus acquiring greater personal relevance (Zator, 2017). These MIPs have also been shown to be effective in increasing PA and decreasing NA when the music is associated with the reminiscence bump (López-Cano et al., 2020).

Visual cues, meanwhile, are not frequently used. This is striking in light of previous findings indicating the dominance of visual stimuli (Schmid et al., 2011) over other types of sensory cues. Indeed, the results of our meta-analysis reveal the greater efficacy of MIPs using pictures to evoke autobiographical memories compared to those using music. This greater effectiveness of visual stimuli could be because (1) the visual modality is predominant over other modalities in attentional and perceptual processes (Sinnett et al., 2007); (2) autobiographical memories tend to be retrieved as mental images (El Haj et al., 2017); (3) some visual stimuli directly correspond with the actual experience of remembered events (Kosslyn et al., 2001); and (4) visual stimuli accelerate and facilitate access to MA content, contributing to MA specificity, vividness, and the realism of the information retrieved (Mazzoni and Memon, 2003; Rubin et al., 2003).

As visual stimuli, the studies included in the present review use impersonal images extracted from the IAPS, personal photographs and photographs of places, all of which prove to be effective in mood repair after a negative mood induction (Carretero et al., 2020). Studies also use film clips (Philippot et al., 2003) and Virtual Reality (Gotz et al., 2022). The lack of works in this line suggests the need to broaden research on such stimuli, given that several studies have found that events evoked by images are reported as more emotional than those elicited by words and even odors (Willander and Larsson, 2006). For this reason, we recommend that future studies employ cues that have thus far been less commonly used. In this sense, visual stimuli are highly powerful cues for accessing the content of AMs and the emotions associated with them, given their close association with the actual experience of the events (e.g., use of personal photographs). Moreover, given that different cues seem to be effective, it is necessary to determine the reasons for their effectiveness. In this line, it would be interesting to analyze not only the type of cue used, but also the quality of the memory to which it facilitates access, taking into account aspects such as the degree of relevance of the memory for an individual, or the extent to which it allows them to relive the past event, since both elements may impact how emotions appear and are experienced.

As for MIPs that use a combination of sensory cues, bimodal procedures are the most common. Some studies employ a combination of verbal and musical cues, with this proving effective in increasing negative emotions such as anger, fear and sadness (Benau and Atchley, 2020), and positive mood (Pacheco-Unguetti and Parmentier, 2014), as well as decreasing negative mood (Pacheco-Unguetti and Parmentier, 2016; Monno et al., 2024). Others combine verbal and visual cues to successfully repair mood (Seebauer et al., 2016). Finally, some studies combine musical and visual cues, proving effective in decreasing NA (Cady et al., 2008). In this sense, it may be considered that the specific coded details of the original event are those that will subsequently allow us to construct autobiographical memories, such that, the more perceptual the cues we use to access the AM, the more effective should be the retrieval of its content. However, to the best of our knowledge, no studies have compared unimodal and bimodal MIPs, and thus this hypothesis cannot be confirmed. Finally, it is worth noting that the results of our meta-analysis show that this type of MIP has a greater effect when the memory-based mood induction follows a prior mood induction (typically of negative mood); in other words, it is used as a method of mood repair (Fernández-Pérez et al., 2023).

Thus, it has been shown that autobiographical-memory-based MIPs, using different types of cues, are effective in inducing mood, both positive and negative. Their effectiveness lies in the recall of past events being a frequent cause of mood variation in daily life (Rimé et al., 1991). Therefore, these are techniques that can be used to study, in laboratory settings, the normal and pathological functioning of mental functions and their relationship with affective states. In addition, the use of neuroimaging techniques could be a successful complement to such techniques.

Regarding the limitations of this work, it should first be noted that the procedures used in the studies included in the review do not take into account the effect of the demand on the generation of the mood state. That is, as shown in previous works (Westermann et al., 1996), participants may discover the goals pursued by the study, regardless of whether or not these are made explicit, and adjust their responses to the experimenter's expectations. Another of the limitations encountered is related to our being unable to include studies that use odors as an autobiographical cue, since none of them met the required quality criteria. This suggests the need to improve the methodology of such procedures in future studies. In addition, the quantitative evidence base is small and unevely reported, which constrained moderator analyses and led to the exclusion of otherwise relevant studies. Most primary studies relied on within-subject pre-post designs and self-report outcomes, with few behavioral or psysiological indices or follow-ups and under non-standardized procedures across laboratories, which limits generalizability and the precision of pooled estimates. To address these issues, future work should preregister and fully report statistics, standardize head-to-head comparisons under common protocols, include multi-method outcomes and longer-term assessments, recruit more diverse and clinical samples, and develop rigorous odor-based AM protocols that also minimize demand characteristics.

Finally, given the diversity of MIPs using autobiographical stimuli, it is necessary to design standardized protocols. This could facilitate their efficacy and replicability. In view of the methodological difficulties detected, we believe future studies should consider the following aspects: (1) the use of equivalent experimental groups; (2) randomly assigning participants to each of the possible experimental conditions; (3) concealing the aim of the mood induction task from participants; (4) controlling for any possible confounding variables that could alter the induction results; (5) the use of standardized measures with good psychometric properties in terms of validity and reliability; (6) the use of objective measures to accompany and enhance the subjective ones; (7) the inclusion of detailed information about the procedure, sample selection, experimental blinding, etc.; (8) measuring mood before and after induction; and (9) including follow-up measures to test the temporal latency of the results of the mood induction procedure.

## 5 Conclusions

The aim of the study was to assess the efficacy of mood induction procedures through accessing evoked autobiographical memories. Our results show that MIPs using autobiographical stimuli of different types (visual, musical, verbal) are effective in inducing emotions, both positive and negative, and even in mood repair. Most MIPs involve a single sensory modality, with the combination of two or more modalities being less common. Generally, the most common method for generating autobiographical memories has been verbal cues, followed by music. In contrast, visual cues have been used less frequently, even though results indicate that this type of cue is highly effective in eliciting autobiographical memories.

However, although scientific literature shows that these MIPs are effective in inducing emotions, the procedures used are very heterogeneous, which makes it difficult to compare the different studies. For this reason, additional research on the relationship between emotions and psychological processes is needed, focusing on method standardization to facilitate comparability across future studies. This constitutes a substantial challenge, which could start by unifying the criteria related, for example, to the use of standardized rating scales for mood states and with which to reliably measure the efficacy of induction.

Given the important role that autobiographical memory plays in mood and in the maintenance of different psychopathologies, knowing which cues (visual, verbal, etc.), individually and/or in combination, and which characteristics of these cues are related to more effective MIPs in inducing positive moods, could help improve the symptoms of certain emotional disorders, such as depression.

## Data Availability

The original contributions presented in the study are included in the article/supplementary material, further inquiries can be directed to the corresponding author.
